# Antioxidant and Anti-Aging Phytoconstituents from *Faucaria tuberculosa*: In Vitro and In Silico Studies

**DOI:** 10.3390/molecules28196895

**Published:** 2023-09-30

**Authors:** Hayam S. Ahmed, Hala Abouzeid, Mostafa A. Mansour, Asmaa I. Owis, Elham Amin, Hany W. Darwish, Ashwag S. Alanazi, Ibrahim A. Naguib, Naglaa Afifi

**Affiliations:** 1 Department of Pharmacognosy, Faculty of Pharmacy, Beni-Suef University, Beni-Suef 62514, Egyptasmaa_owis@yahoo.com (A.I.O.); 2 Department of Pharmacognosy, Faculty of Pharmacy, Port Said University, Port Said 42515, Egypt; 3 Department of Pharmaceutical Chemistry, Faculty of Pharmacy, Nahda University, Beni-Suef 62521, Egypt; 4 Department of Pharmacognosy, Faculty of Pharmacy, Heliopolis University for Sustainable Development, Cairo 11785, Egypt; 5 Department of Medicinal Chemistry and Pharmacognosy, College of Pharmacy, Qassim University, Buraydah 51452, Saudi Arabia; 6 Department of Pharmaceutical Chemistry, College of Pharmacy, King Saud University, Riyadh 11451, Saudi Arabia; 7 Department of Pharmaceutical Sciences, College of Pharmacy, Princess Nourah Bint Abdulrahman University, Riyadh 11671, Saudi Arabia; 8 Department of Pharmaceutical Chemistry, College of Pharmacy, Taif University, P.O. Box 11099, Taif 21944, Saudi Arabia; i.abdelaal@tu.edu.sa

**Keywords:** anti-aging, anioxidant, *Faucaria tuberculosa*, hyaluronidase enzyme, tyrosinase enzyme, molecular docking simulation

## Abstract

Research targeting natural cosmeceuticals is now increasing due to the safety and/or limited side effects of natural products that are highly valued in cosmetology. Within a research program exploring botanical sources for valuable skincare antioxidant components, the current study investigated the phytochemical content and the biological potential of *Faucaria tuberculosa*. Phytochemical investigation of *F. tuberculosa* extract resulted in purification and characterization of six phytoconstituents, including a new one. The structure of the new constituent was elucidated as (-) catechin-(2→1′,4→2′)-phloroglucinol (**4**). The structural identity of all isolated compounds were confirmed on the basis of extensive physical and spectral (1D, 2D-NMR and HRESIMS) investigations. The ethanolic extract exhibits a rich content of total phenolics (TPC) and total flavonoids (TFC), estimated as 32 ± 0.034 mg GAE/g and 43 ± 0.004 mg RE/g, respectively. In addition, the antioxidant (ABTS and FRAP), antihyaluronidase and antityrosinase activities of all purified phytoconstituents were evaluated. The results noted (-) catechin-(2→1′,4→2′) phloroglucinol (**4**) and phloroglucinol (**1**) for their remarkable antioxidant activity, while isorhamnetin 3-*O*-rutinoside (**3**) and 3,5-dihydroxyphenyl *β*-D-glucopyranoside (**2**) achieved the most potent inhibitory activity against tyrosinase (IC_50_ 22.09 ± 0.7 µM and 29.96 ± 0.44 µM, respectively) and hyaluronidase enzymes (IC_50_ 49.30 ± 1.57 µM and 62.58 ± 0.92, respectively) that remarkably exceeds the activity of the standard drugs kojic acid (IC_50_ = 65.21 ± 0.47 µM) and luteolin, (IC_50_ = 116.16 ± 1.69 µM), respectively. A molecular docking study of the two active compounds (**3** and **2**) highlighted their high potential to bind to the active sites of the two enzymes involved in the study.

## 1. Introduction

Aizoaceae is an important plant family predominant mainly in tropical and subtropical areas, and most of its members are ornamental [1]. It includes approximately 135 genera and 2499 species [2]. *Faucaria* is one of these genera, with the subfamily Ruschioideae, known as Tiger jaws due to its appearance [3]. *Faucaria tuberculosa* (Rolfe) Schwant (Syn, F. felina subsp. *tuberculosa* (Rolfe) L.E.Groen, and *Mesembryanthemum tuberculosum* (Rolfe)) have special appeal due to the marked tubercles on the upper surface of the leaves making them look like rough stone [4,5]. Reviewing the relevant literature, no research discussing the phytochemical or the biological activity of this species exists, so the current study was undertaken to explore this plant species.

Skin aging is an undesirable multifaceted process causing unwanted signs, e.g., dryness of the skin, wrinkles, hyper-pigmentation, reduced skin elasticity, and skin cancer. Unwanted side effects and allergic reactions, caused by synthetic drugs, have prompted a search for safe, natural anti-aging products. Aging can be classified as intrinsic and extrinsic. Intrinsic aging might be attributable to genetic or hormonal factors while the extrinsic aging may result from exposure to toxins, chemicals, and UV radiation [6,7,8]. Photo aging is usually accompanied by the formation of reactive oxygen species (ROS). Skin alteration is mediated by many factors including oxidative stress. Accordingly, antioxidants, such as polyphenols, could be an effective treatment that reduces oxidative stress and improves skin condition [9]. The aging process is also associated with increasing enzyme activity as tyrosinase and hyaluronidase [7]. Tyrosinase enzymes regulate the process of melanin synthesis, and melanin accumulation leads to several skin ailments, such as wrinkles, brown spots, age spots, etc. [10]. Hyaluronic acid is a substance produced naturally by the body to preserve moisture content in skin tissues as well as in other organs, and hyaluronidase is the enzyme responsible for hyaluronic acid degradation [11].

In vitro studies assessing the antioxidant and antiaging activity of Aizoaceae extracts were previously reported, in which extracts of *Mesembryanthemum Nodiflorum*, *Mesembryanthemum crystallinum*, *Mesembryanthemum forsskaolii*, *Carpobrotus edulis* and *Mesembryanthemum edule* were reported to possess a concentration-dependent free radical scavenging activity against DPPH and hydrogen peroxide radicals, which was comparable with standard ascorbic acid [12]. Additionally, *Carpobrotus edulis* aqueous leaf extract was reported to increase wound closure, collagen production, and inhibit collagenase and hyaluronidase [13]. In cell culture experiments, CAE increased wound closure and collagen production, which was consistent with its high polyphenol content. Many pure compounds from different chemical classes (e.g., alkaloids, flavonoids, steroids) have been isolated from Aizoaceae plants and were found responsible for their multiple therapeutic activities, such as antioxidant, anti-inflammatory, antihepatotoxic, anticancer, and antimicrobial activities [14]. Since *F. tuberculosa* has not been previously investigated phytochemically and/or biologically, and preliminary studies have shown promising contents of total phenolics and flavonoids, the authors conducted this study with regard to the isolation of secondary metabolites and evaluation of their antioxidant potential, anti-tyrosinase, and anti-hyaluronidase properties. Firstly, an in vitro model was designed to identify the compound(s) with the highest anti-aging activity; secondly, in silico models were applied to the most active anti-aging compound on the two enzymes.

## 2. Results and Discussion

### 2.1. Evaluation of TPC and TFC of Ethanolic Extract

TPC was quantitated using Folin–Ciocalteu reagent, while aluminium chloride was used to determine TFC in *F. tuberculosa* ethanolic extract. The current findings calculate TPC and TFC as 32 ± 0.034 mg GAE/g and 43 ± 0.004 mg RE/g, respectively. These results indicated an appreciable content of phenolics and flavonoids in this species, which may make it promising in terms of biological activities. It worth mentioning that this is the first study investigating the phytoconstituents contents in genus *Faucaria*.

### 2.2. Spectroscopic Data of Isolated Compounds

**Figure 1:**
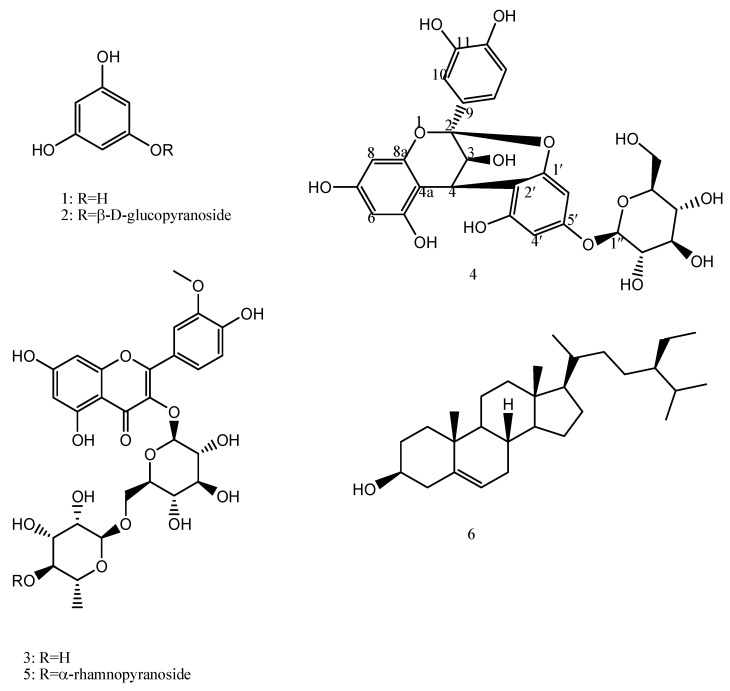
Structures of the phytoconstituents (1–6) from *F. tuberculosa*.

Chromatographic fractionation of the polar fractions of *F. tuberculosa* afforded five compounds (Figure 1); phloroglucinol (**1**), 3,5-dihydroxyphenyl *β*-D-glucopyranoside (Phlorin; **2**), isorhamnetin 3-*O*-rutinoside (**3**), (-) catechin-(2→1′,4→2′)-phloroglucinol (**4**), and isorhamnetin 3-*O*-[α-rhamnopyranosyl-(1→4)-*α*-rhamnopyranosyl-(1→6)-*β*-glucopyranoside (**5**). *β*-sitosterol (**6**) was purified from the non-polar fraction. Herein, compound **4** is reported as a new natural compound, while other phytoconstituents are isolated for the first time from *F. tuberculosa*.

Interestingly, the current study reports the isolation of compound **4** from nature for the first time. The structural elucidation of **4** was performed using physical and spectrophotometric studies. Other known compounds were identified based on their NMR spectral data, and confirmed by comparison with the relevant data reported in the literature [15,16,17,18,19,20].

Compound **4** was isolated as a pale brown amorphous powder. It becomes an orange colour in response to *p*-anisaldehyde-sulphuric acid spray reagent, a colour characteristic of proanthocyanidin compounds [21]. It displays peaks at 224 and 278 nm in the UV spectrum, [α]_D_^25^ = −9 (MeOH), and a molecular ion peak, [M-H]^−^, with *m/z* 573.1323 recorded in the HRMS spectrum (Figure S7).

**Figure 2:**
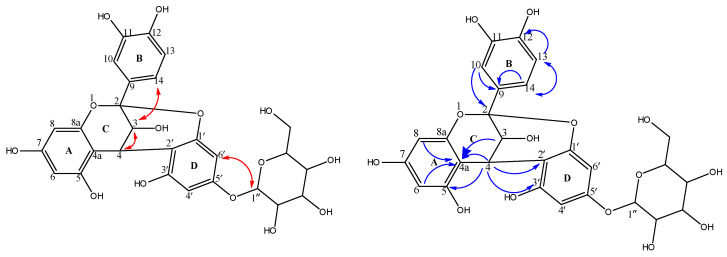
Key HMBC (blue colour) and NOESY (red colour) correlations.

^1^H-NMR (Table 1, Figure S8) displayed signals at δ_H_ 4.06 (d, *J* = 3.6 Hz; H-3), 4.65 (d, *J* = 4 Hz; H-4), representing an AB system, in addition to an AMX coupling system: δ_H_ 7.13 (d, *J* = 2.4, H-10), 6.83 (d, *J* = 2.4, H-13), 7.01 (dd, *J* = 2, 8.2, H-14). In the ^13^C NMR and HSQC spectra (Figures S9 and S10), the presence of signals at δ_C_ 100.3, 68.08 and 29.5 is attributed to the heterocyclic C-2, C-3, and C-4 of ring C. The aromatic A-, B- and D-ring carbons were observed downfield at δ_C_104.5 (4a), 155.5(5), 98.6(6), 156.2(7), 96.9(8), 158.6(8a), 132.1(9), 115.65(10), 146.7(11), 145.7(12), 115.67(13) and 119.8(14). These spectral data were similar to data for the upper flavanol moiety of the previously reported proanthocyanidin A-2; epicatechin-(2*β*→7,4*β*→8)-epicatechin [21]; however, NOE data were recorded to confirm cis or trans configuration of H-3, H-4 and H-14, as indicated below. Proanthocyanidins are oligomers or polymers composed of flavanols units. They are classified into subclasses; among them, the A-type is a subclass characterized by a double linkage between two consecutive flavanol moieties. One of them is a single 4C-8D or 4C-6D bond, while the other is ether-type bond between 2C-*O*-7D or 2C-*O*-5D [22,23]. NMR spectral data for compound **4** differ from proanthocyanidin A-2 in the absence of signals of a lower flavonol moiety. As indicated in Table 1, both ^1^H- and ^13^C-NMR data showed characteristic resonances for a 1, 2 disubstituted phloroglucinol moiety (δ_C_108.2, 154.4, 98.1, 154.9, 96.8, 158) and a *β*-glucopyranoside moiety (δ_C_ 101.9, 74.6, 77.3, 71.2, 78.2, 62.3). HMBC (Table 1, Figure S11) showed ^2^*J*_C-H_ correlation between H-4 and C-4a and ^3^*J*_C-H_ correlation between H-4 and, C-2, C-2′, C-3′ confirming the linkage between the flavanol and phloroglucinol moieties. This double-linked skeleton was confirmed by presence of one acetal carbon at δ_C_ 100.3 (C-2) and one quaternary carbon at δ_C_ 108.2 (C-2′) in its ^13^C NMR spectrum. The identities of the sugar units were elucidated via comparison of their ^13^C NMR resonances with the literature [17,24]. The anomeric proton exhibited a *J* value of 7.6 Hz, thus confirming *β*-type glycosidic linkage [17,24]. The position of the glucopyranoside moiety was elucidated in the HMBC spectrum, as it displayed a ^3^*J*_C-H_ correlation between the anomeric proton of the glucose moiety at δ_H_ 4.97(d, 7.6, H-1″) to 5′ at δ_C_ 154.9 ppm. Based on their similarity with A-type proanthocyanidins, H-3 and H-4 cis or trans orientation cannot be distinguished based on coupling constants that record very similar values. However, they can be determined via NOE correlation (Figures S12 and S13). H-3 showed a NOE interaction with H-14; a NOE interaction, characteristic of a 3, 4-trans orientation, was not detected between H-3C with H-6D. Accordingly, the interaction between H-3C and H-4C is cis, and independently of the rigid ring conformation, the flavanol moiety will be catechin [23,25]. Accordingly, compound **4** was concluded as (-) catechin-(2→1′,4→2′)-phloroglucinol (Figure 1 and Figure 2), which was isolated as a natural product herein for the first time.

### 2.3. Antioxidant Activities of Isolated Compounds

ROS are regularly generated in most living tissues. They can damage DNA, proteins and lipids at high concentrations. Several studies have reported that ROS plays a key role in the mechanism of aging [26,27,28]. To evaluate the antioxidant activity of the isolated compounds (**1**–**6**), we examined their ABTS^+^ radical scavenging capacity, as well as the total reducing power (FRAP). The antioxidant activity of phenolics is affected by their chemical structures; OH groups represent a key factor regulating the ability of these compounds to scavenge the free radicals hydroxyl [29,30]. As indicated in Table 2, the two compounds catechin-(2→1′,4→2′)-phloroglucinol (**4**) and phloroglucinol (**1**) exhibited strong ABTS radical scavenging activity with IC_50_ values of 4.11 ± 0.32 and 6.44 ± 0.47 µg/mL, respectively, which seems better that the standard used (ascorbic acid). Additionally, compounds **3**, **5** and **2** showed good antioxidant, while **6** was the least active among the tested compounds. Interestingly, the results of the ferric reducing antioxidant power (FRAP) assay were in great accordance with the ABTS radical scavenging assay results, wherein catechin-(2→1′,4→2′)-phloroglucinol and phloroglucinol showed the strongest reducing power, with IC_50_ values better that ascorbic acid (Table 2). The strong antioxidant activity of catechin-(2→1′,4→2′)-phloroglucinol may be returned to the linkage between the flavan and phloroglucinol moieties, as well as the hydroxyl groups after the pattern of A-type proanthocyanidin [30]. The strong ABTS radical scavenging activity of phloroglucinol was consistent with the previously reported data [31]. This remarkable antioxidant activity was attributed to the presence of acidic hydrogen [32]. Compound **5** exhibited antioxidant and reducing powers, with an IC_50_ lower than compound **3**. The decrease in the activity of both flavonoids may be related to their glycosylation at C-3 [33].

The moderate activity of phlorin (**2**) (when compared with phloroglucinol) also matched the reported DPPH radical scavenging activity of its butanoyl derivative [34]. Hence, presence of more sugar moieties in phlorin (**2**) and isorhamnetin glycoside (**5**) causes a reduction in their antioxidant activity. *β*-sitosterol showed weak ABTS activity and no reducing antioxidant power (FRAP). These results matched those in previous reports of this compound [35].

### 2.4. Anti-Aging Activities of Isolated Compounds

#### 2.4.1. Tyrosinase Inhibitory Assay

**Figure 3:**
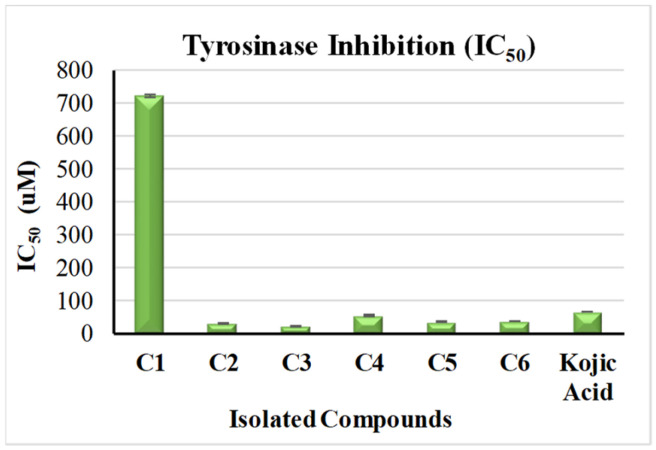
Anti-tyrosinase activity of the phytoconstituents from *F. tuberculosa*: (**C1**) phloroglucinol, (**C2**) phlorin, (**C3**) isorhamnetin 3-*O*-rutinoside, (**C4**) (-) catechin-(2→1′,4→2′)-phloroglucinol, (**C5**) isorhamnetin 3-*O*-[*α*-rhamnopyranosyl-(1→4)-*α*-rhamnopyranosyl-(1→6)-*β*-glucopyranoside], (**C6**) *β*-sitosterol.

Tyrosinase played an important role in melanin biosynthesis, which in turn leads to age spots [36]. In our study, all the isolated compounds were investigated in vitro for the inhibitory activities of tyrosinase enzymes, using kojic acid (IC_50_ = 65.21 ± 0.47 µM) standard. As demonstrated in Figure 3, isorhamnetin 3-*O*-rutinoside (**3**) and phlorin (**2**) exhibited an extremely potent tyrosinase inhibition, with IC_50_ values of 22.09 ± 0.7 µM and 29.96 ± 0.44 µM, respectively, followed by compounds **4**, **5** and **6,** which also showed strong activity with IC_50_ values of 54.47 ± 1.59 µM, 34.61 ± 1.36 µM and 36.99 ± 0.78 µM, respectively. On the other hand, compound **1** showed very weak activity, with an IC_50_ of 722.940 ± 4.64 µM. Remarkably, the current study is the first study that investigates the anti-tyrosinase activity of compounds **1**–**5**. Compound **6** was previously noted for its significant binding affinity with tyrosinase enzymes [36].

#### 2.4.2. Hyaluronidase Inhibitory Assay

**Figure 4:**
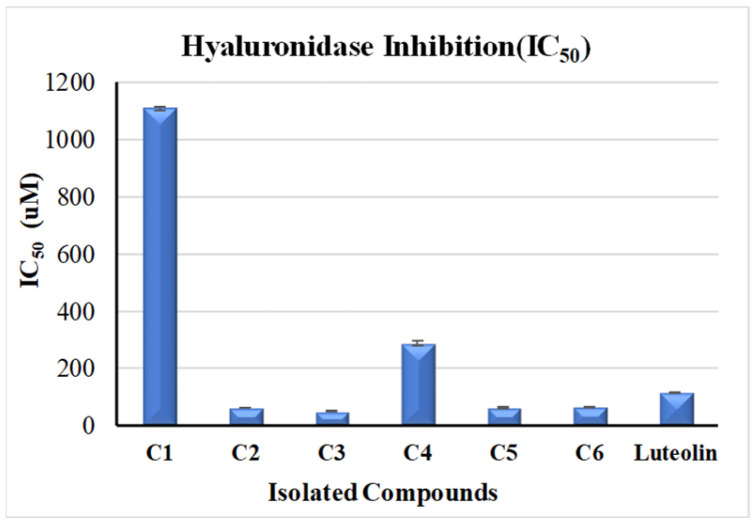
Anti-hyaluronidase activity of the phytoconstituents from *F. tuberculosa*: (**C1**) phloroglucinol, (**C2**) phlorin, (**C3**) isorhamnetin 3-*O*-rutinoside, (**C4**) (-) catechin-(2→1′,4→2′)-phloroglucinol, (**C5**) isorhamnetin 3-*O*-[*α*-rhamnopyranosyl-(1→4)-*α*-rhamnopyranosyl-(1→6)-*β*-glucopyranoside], (**C6**) *β*-sitosterol.

In vitro investigation of the hyaluronidase inhibitory potential of the isolated compounds, using luteolin (IC_50_ = 116.16 ± 1.69 µM) as a standard, indicated similar results to those of the anti-tyrosinase activity testing (Figure 4). Isorhamnetin 3-*O*-rutinoside (**3**) showed the most potent activity, followed by phlorin (**2**), with IC_50_ values of 49.3 ± 1.57 µM and 62.58 ± 0.92 µM, respectively. Additionally, compounds **5** and **6** showed good activity, with IC_50_ values of 63.31 ± 2.84 µM and 65.45 ± 1.38 µM. Notably, all the four compounds displayed better activity than the standard used. Additionally, (-) catechin-(2→1′,4→2′)-phloroglucinol (**4**) displayed medium inhibitory activity (IC_50_ 287.39 ± 8.4 µM), while phloroglucinol (**1**) showed very weak activity, with IC_50_ values of 1108.56 ± 7.11 µM.

### 2.5. In Silico Molecular Docking

#### 2.5.1. Anti-Tyrosinase Activity

**Figure 5:**
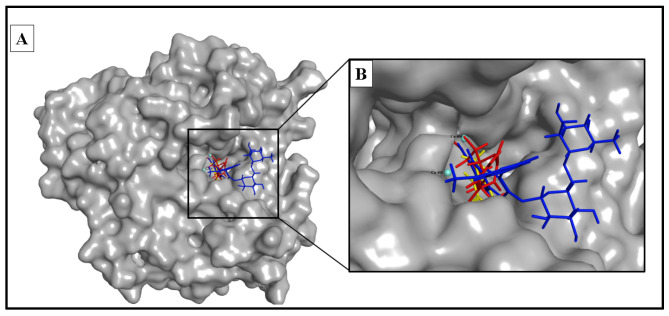
Phlorin, isorhamnetin 3-*O*-rutinoside and kojic acid bound to the active domain of tyrosinase enzyme (PDB: 2Y9X): (**A**) 3D binding presentation of tyrosinase enzyme; (**B**) magnified 3D binding mode showing the active site of a tyrosinase enzyme containing two copper ions and the three docked ligands (phlorinin in red, isorhamnetin 3-*O*-rutinoside in blue, and kojic acid in yellow). The figure was generated using BIOVIA Discovery Studio 2021.

**Figure 6:**
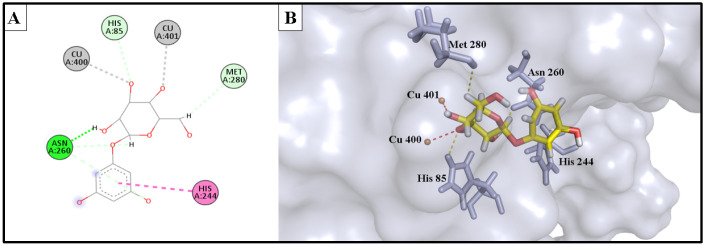
(**A**) 2D interaction diagram of the top docking pose of phlorin into the active site of the tyrosinase enzyme; (**B**) 3D interaction diagram of the top docking pose of phlorin into the active site of the tyrosinase enzyme.

**Figure 7:**
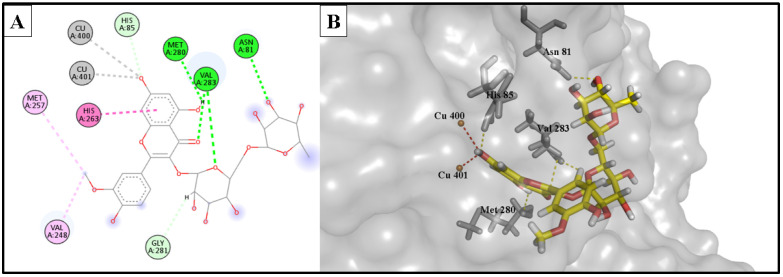
(**A**) Two-dimensional interaction diagram of the top docking pose of isorhamnetin 3-*O*-rutinoside into the active site of the tyrosinase enzyme; (**B**) Three-dimensional interaction diagram of the top docking pose of isorhamnetin 3-*O*-rutinoside into the active site of the tyrosinase enzyme.

**Figure 8:**
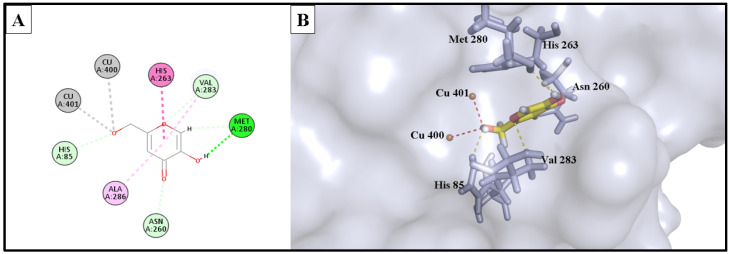
(**A**) Two-dimensional interaction diagram of the top docking pose of kojic acid into the active site of the tyrosinase enzyme; (**B**) Three-dimensional interaction diagram of the top docking pose of kojic acid into the active site of the tyrosinase enzyme.

The docking study involved simulation of the most active compounds, isorhamnetin 3-*O*-rutinoside (**3**) and phlorin (**2**), compared to the current inhibitor (kojic acid), in the tyrosinase cleavage site (PDB ID: 2Y9X). Figure 5 shows the binding site of the three compounds in the binuclear copper-containing domain. We compared the optimal binding poses of phlorin (**2**) and isorhamnetin 3-*O*-rutinoside (**3**) with that of the well-known tyrosinase inhibitor kojic acid. Figure 6, Figure 7 and Figure 8, respectively, depict the most stable binding poses based on the MOE 2022.01 scoring (described in Table 3) within the catalytic domain of tyrosinase (2D and 3D interaction captions).

The demonstrated molecular docking results nicely matched with the in vitro testing results, wherein isorhamnetin 3-*O*-rutinoside (**3**) showed the highest binding score (with a -12.154 kcal/mol binding energy) to the binuclear copper ions of the catalytic domain, with strong metal acceptor bonds to the copper ions. Moreover, it showed strong H-bond acceptance to Met 280 amino acid; also, it formed stable H-bond acceptor bonds with His 85, Val 283, and Asn 81 residues, in addition to many hydrophobic interactions within the binding site of the tyrosinase enzyme. These strong interactions as well as the bulkiness of isorhamnetin 3-*O*-rutinoside could provide an acceptable explanation of the strong enzyme assay results of this compound against the tyrosinase enzyme. Similarly, phlorin (**2**) showed a strong binding interaction, with a binding affinity of −10.953 kcal/mol, and served as strong metal acceptor of the binuclear copper ions of the catalytic domain. Additionally, it demonstrated an H-bond acceptor interaction with His 85 amino acid, which seems an essential binding residue for this activity. Moreover, it interacted via H-bond donor bonds with Asn 260 and Met 280. In addition, it exhibited hydrophobic interactions with His 244 residue. These strong interactions may explain and match with the enzyme assay results of this compound against tyrosinase enzyme.

On the other hand, the positive control and the reference drug kojic acid showed a strong binding interaction with the active domain of the tyrosinase enzyme, with a binding affinity of −9.385 kcal/mol. It served as strong metal acceptor, and formed strong bonds to copper ions incorporated at the active site. Kojic acid also showed an H-bond acceptor interaction with His 85 residues, which validates the importance of these amino acids as inhibitors of the tyrosinase enzyme. Kojic acid also bound to Asn 260 and Met 280 residues with H-bond acceptor and H-bond donor interactions, respectively, and showed a hydrophobic interaction with His 263 amino acid and served as an H-bond acceptor for Val 283 residues.

#### 2.5.2. Anti-Hyaluronidase Activity

**Figure 9:**
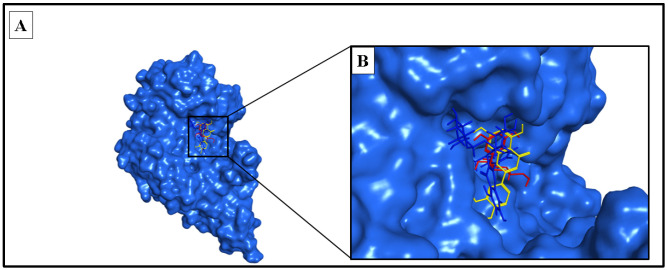
Phlorin, isorhamnetin 3-*O*-rutinoside and luteolin bound to the active domain of the hyaluronidase enzyme (PDB: 1FCV): (**A**) 3D binding presentation of the hyaluronidase enzyme; (**B**) magnified 3D binding mode showing active site of the hyaluronidase enzyme containing the three docked ligands (phlorin in red, isorhamnetin 3-*O*-rutinoside in blue, and luteolin in yellow). The figure was generated using BIOVIA Discovery Studio 2021.

**Figure 10:**
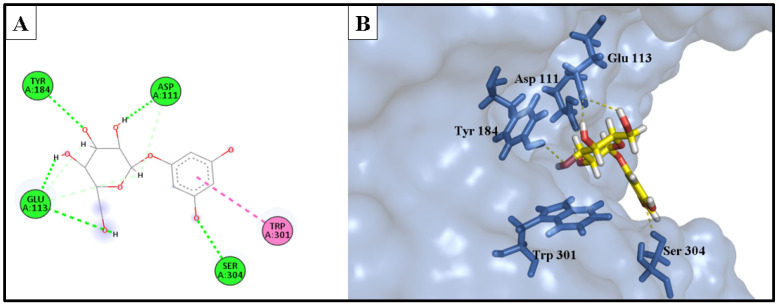
(**A**) Two-dimensional interaction diagram of the top docking pose of phlorin into the active site of the hyaluronidase enzyme; (**B**) Three-dimensional interaction diagram of the top docking pose of phlorin into the active site of the hyaluronidase enzyme.

**Figure 11:**
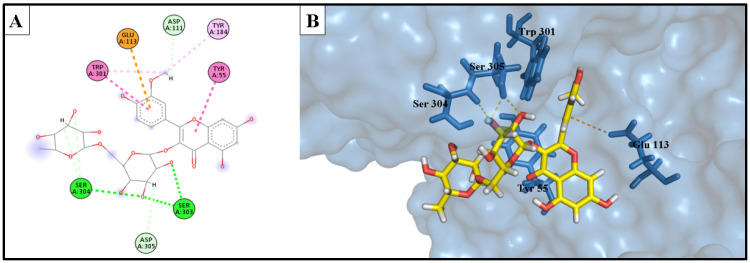
(**A**) Two-dimensional interaction diagram of the top docking pose of isorhamnetin 3-*O*-rutinoside into the active site of the hyaluronidase enzyme; (**B**) Three-dimensional interaction diagram of the top docking pose of F3 into the active site of the hyaluronidase enzyme.

**Figure 12:**
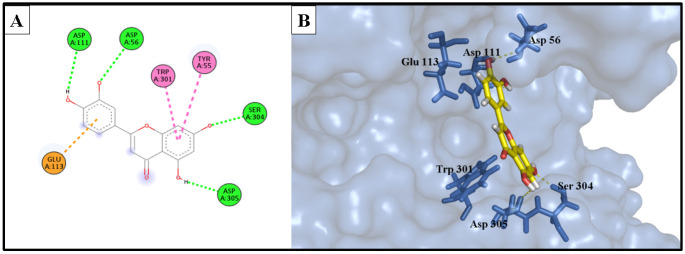
(**A**) Two-dimensional interaction diagram of the top docking pose of luteolin into the active site of the hyaluronidase enzyme; (**B**) Three-dimensional interaction diagram of the top docking pose of luteolin into the active site of the hyaluronidase enzyme.

The docking investigation involved simulation of the most potent compounds isorhamnetin 3-*O*-rutinoside (**3**) and phlorin (**2**), compared to the current inhibitor luteolin, into the active site of hyaluronidase (PDB ID: 1FCV). Figure 9 shows the binding site of both compounds within the catalytic domain of the hyaluronidase enzyme. Comparing the optimal binding poses of isorhamnetin 3-*O*-rutinoside and phlorin with those of the positive control, luteolin, Figure 10, Figure 11 and Figure 12 depict the most stable binding poses based on the scoring method of the docking machine (described in Table 3) within the catalytic domain of hyaluronidase (2D and 3D interaction captions).

Isorhamnetin 3-*O*-rutinoside (**3**) showed a very strong interaction with the binding site of the hyaluronidase enzyme, with a −16.578 kcal/mol binding affinity. It exhibited a strong H-bond donor interaction with Asp 111 amino acid, H-bond acceptor interactions with Ser 304 amino acid, and two significant interactions with Ser 303 amino acid, as well as a unique electrostatic interaction with the amino acid Glu 113. Furthermore, a lot of hydrophobic interactions with the catalytic domain of the hyaluronidase enzyme were also displayed.

Phlorin (**2**), the second most active constituent, showed a strong binding interaction with the active domain of the hyaluronidase enzyme, with a binding affinity of −14.979 kcal/mol. It showed excellent H-bond donor interaction, with an Å bond distance of 1.91, with the Asp 111 residue. It exhibited a bidentate H-bond donor interaction with Glu 113 amino acid. Additionally, it formed H-bond acceptor interactions with Ser 304 and Tyr 184 amino acids, and a hydrophobic interaction between the phenyl ring and Trp 301 residue.

These strong interactions notably account for the recorded in vitro anti-enzyme activity of isorhamnetin 3-*O*-rutinoside (**3**) and phlorin (**2**) against the hyaluronidase enzyme.

The reference drug, luteolin, showed a relativity weaker interaction to the active domain of the enzyme, with a binding affinity of −12.404 kcal/mol, if compared to the binding affinities of isorhamnetin 3-*O*-rutinoside (**3**) and phlorin (**2**), which may explain the superior activity of these compounds compared with the positive control luteolin. The binding interactions of luteolin were involved in two H-bond donor interactions with Asp 111 and Asp 305 residues. In addition, it formed three hydrophobic interactions with Glu 113, Trp 301, and Tyr 55 amino acids; it served as an H-bond acceptor for Ser 304 at the edge of the hyaluronidase active domain, and also for Asp 56 residue with the *m*-OH of the phenyl ring.

## 3. Materials and Methods

### 3.1. Plant Material and Extraction

*F. tuberculosis* (aerial parts) were collected in September 2020 from El-Hosary public garden in 6th October City, Egypt. The plant’s identity was taxonomically confirmed by Ms. Trease Labib, El-Orman Botanical Garden, Giza, Egypt. A plant sample (with voucher number BUPD-118) was preserved at the College of Pharmacy, BSU, Egypt. *F. tuberculosis* (750 g, fresh aerial parts) was steeped in ethanol at RT and filtered, and the filtrate was dried under a vacuum to yield a 150 g residue, which was used for evaluation of the total antioxidant activity, TPC, TFC, and chromatographic isolation. For chromatographic isolation, the residue was partitioned using different solvents with sequential polarities; the obtained fractions were then dried under a vacuum.

### 3.2. General Experimental Materials and Procedures

Silica gel 60 (particle size 0.063–0.2 mm, 70–230 mesh) (Fluka, St. Louis, MO, USA), Polyamide-6 and Sephadex LH-20 (Sigma-Aldrich, Taufkirchen, Germany) were utilized for column chromatography. TLC plates (Si 60 F_254_, Merck, Darmstadt, Germany) and analytical-grade solvents were used in the study. Visualization of the spots was carried out using *p*-anisaldehyde spray reagent [37].

The UV investigation was carried out in methanol utilizing a Shimadzu UV1, 601PC UV–visible scanning spectrophotometer (Shimadzu Corp., Tokyo, Japan). Optical rotation was measured using a 341 Perkin Elmer polarimeter (Darmstadt, Germany). HRESIMS was executed on Agilent LC/Q-TOF, 6530 (Santa Clara, CA, USA). All 1D and 2D NMR spectra were recorded on a Bruker Avance III 400 MHz (Bruker AG, Fällanden, Switzerland) and analysed using Topspin 3.1 software (Bruker AG, Fällanden, Switzerland). Deuterated methanol and chloroform (Cambridge Isotopes, Andover, MA, USA) were used.

Aluminium trichloride (AlCl_3_.6H_2_O), anhydrous sodium carbonate (Na_2_CO_3_), ascorbic acid, DPPH, ABTS (Sigma, PN: A3219, St. Louis, MO, USA), Folin–Ciocalteu, gallic acid, rutin, sodium hydroxide (NaOH), and sodium nitrite (NaNO_2_) were purchased from Merck (Rahway, NJ, USA) and Sigma-Aldrich (St. Louis, MO, USA).

### 3.3. Evaluation of the Total Phenolic Content (TPC) of Ethanolic Extract

The total phenolics in the crude extract was assessed using Folin–Ciocalteu’s reagent according to Jimoh et al., with slight modifications [38]. Ethanol extract (0.5 mL) and 2.25 mL of Folin–Ciocalteu reagent (10% aqueous solution) were mixed, and 5 min later, 2.25 mL of Na_2_CO_3_ (7.5%, *w*/*v*) was added; the solution was kept for 30 min, and absorbance was then measured at 725 nm using a spectrophotometer. The results were calculated as mg gallic acid equivalents per 1 g of dried extract (mg GAE/g).

### 3.4. Evaluation of the Total Flavonoid Content (TFC) of Ethanolic Extract

Flavonoids contents were measured using the modified colorimetric method. [39] In a test tube, diluted extract (0.5 mL extract + 2.25 mL distilled water) was mixed with 0.15 mL of 5% NaNO_2_ solution, and 6 min later, AlCl_3_.6H_2_O solution (10%, 0.3 mL) was added; the solution was kept for 5 min, and then 1.0 mL of 1 M NaOH was added. The absorbance was then measured at 510 nm using a spectrophotometer. TPC was estimated as mg rutin equivalents per 1 g of dried extract (mg RE/g).

### 3.5. Chromatographic Isolation of Phytoconstituents

TLC screening of the different fractions revealed similar spots in both ethyl acetate (EtOAc) and butanol (*n*-BuOH) fractions; accordingly, both fractions were combined. The pooled fraction (2.2 g) was fractionated on a polyamide-6 column (80 × 2.8 cm, i.d) with an aqueous methanol gradient to obtain F-I, F-II, and F-III. Fraction F-I (700 mg, eluted with 5% aq. MeOH) was applied on an Si column, using elution with CH_2_Cl_2_ containing increasing proportions of MeOH to attain the sub fractions F-Ia (30 mg) and F-Ib (40 mg). Both fractions were re-chromatographed using Sephadex LH-20 c.c. and eluted with MeOH to isolate compounds **1** (10 mg) and **2** (12 mg). F-II (50 mg, 70% aq. MeOH) was similarly treated to isolate compound **3** (8 mg). Additionally, F-III (50 mg, 90% aq. MeOH) was subjected to the same procedure to obtain compounds **4** (8 mg) and **5** (10 mg).

*n*-hexane extract was subjected to gradient chromatography on a Si gel column, using n-hexane-EtOAc mixtures as the mobile phase, to obtain compound **6** (5 mg).

The NMR data for compounds **1**, **2**, **3**, **5** and **6** are included in the supplementary data.

### 3.6. Antioxidant Activities of the Isolated Compounds

The antioxidant activity of the isolated compounds was determined using ABTS and FRAP assays in triplicate, and the average values were considered.

#### 3.6.1. ABTS Radical Scavenging

This is a decolorization assay method in which the antioxidant capacity of the isolated compounds is measured via a reaction with an ABTS cation radical; we adopted the method described by others [40,41,42]. To produce the ABTS radical, the stock solution of ABTS (1.8 mM) was reacted with potassium persulfate (0.63 mM), and the mixture was kept in the dark, at RT, for 12–16 h. The reaction mixture was then adjusted to obtain 0.700 (±0.030) absorbance at 734 nm via dilution with ethyl alcohol. Afterwards, the radical solution (190 μL) and sample solution (10 μL) were mixed, and the absorbance was recorded regularly, at 734 nm, every minute up to a time period of 13 min from initial mixing. Ascorbic acid (standard drug) and 80% methanol (negative control) were similarly treated as the sample solution.

#### 3.6.2. Ferric Reducing/Antioxidant Power (FRAP)

Herein, antioxidant potential was measured based on the samples’ ability to reduce ferric to ferrous ion [43]. This method relies on the reduction of ferricyanide relative to the sample. Samples (1 mL) were mixed with 2.5 mL of each of sodium phosphate buffer and K_3_Fe (CN)_6_, and then the reaction mixture was incubated (50 °C, 20 min). Afterward, 2.5 mL of trichloroacetic acid was added, and centrifugation (1000× *g*, 10 min) was carried out. Some 2.5 mL of the supernatant solution and an equal amount of deionized H_2_O and 0.5 mL of ferric chloride were then mixed, and the absorbance was recorded at 700 nm.

### 3.7. Anti-Aging Activities of the Isolated Compounds

The anti-aging potential of the *F. tuberculosa* phytoconstituents was assessed via evaluation of their anti-tyrosinase and anti-hyaluronidase activities. Both assays were carried out in triplicate, and average values were considered.

#### 3.7.1. Tyrosinase Inhibitory Assay

To measure tyrosinase activity, a colorimetric assay was performed by using Tyrosinase Inhibitor Screening Kit (# K575-100, BioVision^®^ Inc., Milpitas, CA, USA), adopting instructions provided by the manufacturer. Firstly, we added 20 μL of the test inhibitors in serial dilutions (Sample, S), an inhibitor control (IC), and tyrosinase assay buffer into wells (enzyme control, EC). Tyrosinase enzyme solution (50 μL) was added to each well, and incubated at 25 °C for 10 min. Then, the tyrosinase substrate solution (30 µL) was added, and the absorbance was recorded at 510 nm for 30–60 min [44]. The % relative inhibition was calculated as follows:

% Relative inhibition = [(Slope of EC − Slope of S) / Slope of EC] × 100(1)

#### 3.7.2. Hyaluronidase Inhibitory Assay

This assay was performed by adopting a turbidimetric assay using a QuantiChrom™ Hyaluronidase Inhibitor Screening Assay Kit. Bovine hyaluronidase (Calzyme Cat # 091A0300) was mixed with the enzyme buffer until a concentration of 10 U/mL was reached. Then, 40 µL of enzyme solution (sample well), enzyme buffer (NEC), and hyaluronidase (used for NIC) were transferred to a 96-well plate. DMSO (20 μL) was added to the NIC and NEC wells, while 20 µL samples were added to the sample wells. The plate was then incubated (15 min, RT). Afterwards, 40 µL of the substrate was added, followed by 20 min incubation. Finally, 160 µL of the stop reagent was added, and a further 10 min of incubation was performed; the optical density was then measured at 600 nm [45].

% Inhibition = [1 − (OD_NEC_ − OD_Test Cpd_) / (OD_No Enzyme_ − OD_NIC_)] × 100 (2)

where OD is the optical density value, NEC refers to no enzyme control, and NIC refers to no inhibitor control.

### 3.8. In Silico Molecular Docking Studies

#### 3.8.1. Anti-Tyrosinase Activity

**Figure 13:**
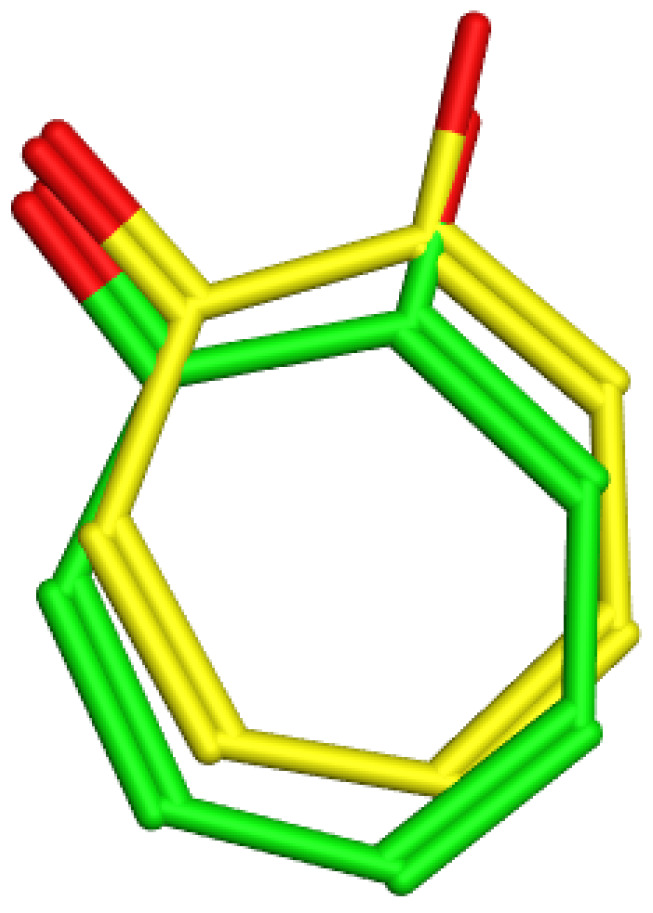
Tyrosinase enzyme: co-crystallized (light green) and re-docked (yellow) ligands’ superimposition.

For the docking study, the crystal structure of *Agaricus bisporus* tyrosinase (PDB code: 2Y9X) [46,47] was downloaded from the protein databank (PDB). Then, the structures of the most active compounds isorhamnetin 3-*O*-rutinoside and phlorin versus the positive control, kojic acid, were drawn with Marvin Sketch (Chem Axon, Boston, MA, USA) [48] and minimized with the MOE 2022.01 (Chemical Computing Group, Montreal, QC, Canada) Amber10:EHT force field general energy minimizing tool (Gradient RMS < 0.1 kcal/mol/Å^2^) [49]. The docking methods and parameters were validated by redocking the native ligand into its active site to ensure that the ligand orientations and positions derived from docking studies were likely to represent valid and reasonable potential binding modes of the inhibitors. The root mean square deviation (rmsd) between the re-docked native ligand and the co-crystallized ligand was 0.5478, as shown in Figure 13.

The most active compounds (**3**) and (**2**) and kojic acid were docked into the active domain of tyrosinase. Fifty poses of each compound were scored using the initial rescoring methodology (London dG) and the final rescoring methodology (London dG) after placement using Triangle Matcher. The post-placement refinement was performed using Force Field [50]. The 2D diagrams of the interactions between ligands and amino acid residues were generated using the free BIOVIA Discovery Studio Visualizer 2021 [51], while the 3D captions were generated using the PyMOL (v0.99) program [52].

#### 3.8.2. Anti-Hyaluronidase Activity

Anti-hyaluronidase docking experiments were carried out using the crystal structure of bee venom hyaluronidase in complex with a hyaluronic acid tetramer (PDB code: **1FCV**) [45,53]. The chemical structures of the most active compounds, isorhamnetin 3-*O*-rutinoside and phlorin, and the reference drug, luteolin, as a well-known flavone [54,55], were drawn and minimized, as in a previous study [48,49]. In the active domain of hyaluronidase, the most active compounds (**3**), (**2**), and luteolin were docked. Fifty poses of each compound were scored using the initial rescoring method (London dG), the final rescoring method (GVBI/WSA dG) after placement using Triangle Matcher; the post-placement refinement was carried out using Force Field [56]. Additionally, the free program BIOVIA Discovery Studio Visualizer 2021 was used to generate the 2D diagrams illustrating the interaction between ligands and amino acid residues [51], while PyMOL (v0.99) was employed in the generation of the 3D captions [52].

## 4. Conclusions

The phytochemical study of *F. tuberculosa* resulted in the isolation of one new in addition to five known compounds. This is the first report of the isolation of these six constituents from the genus *Faucaria*. All compounds, except for *β*-sitosterol, displayed a remarkable antioxidant activity, as observed using ABTS and FRAP assay methods. Isorhamnetin 3-*O*-rutinoside and phlorin were highlighted for their potent inhibitory activity against both tyrosinase and hyaluronidase enzymes using both in vitro and in silico testing. Overall, the obtained results support the applicability of these compounds as pigmentation modulators and means of skin remodelling in the pharmaceutical industry.

## Figures and Tables

**Table 1:** NMR spectroscopic data of compound 4.

Position	δ_H_ (mult, J)	δ_C_	HMBC (H→C)
Flavanol moiety			
2	----	100.3	----
3	4.05 (d, 3.6)	68.08	C-4a
4	4.64 (d, 4)	29.5	C-4a, C-2, C-2′, C-3′
4a	----	104.5	----
5	----	155.5	----
6	6.03(d, 2.4)	98.6	C-4a
7	----	156.2	----
8	6.10 (d, 2.4)	96.9	----
8a	----	158.6	-----
9	----	132.1	-----
10	7.13 (d, 2.4)	115.65	C-2, C-9, C-11, C-12, C-14
11	----	146.7	----
12	----	145.7	----
13	6.81 (d, 8.4)	115.67	C-2, C-11, C-12, C-14
14	7.01 (dd, 2, 8.4)	119.8	C-2, C-9, C-10, C-12, C-13
Phloroglucinol moiety			
2′	----	108.1	
3′	----	154.5	
4′	6.08 (d, 2)	98.1	C-2′, C-5′
5′	----	154.9	
6′	6.23 (d, 2.4)	96.8	C-1′, C-2′
1′	----	158.1	----
*β*-glucopyranoside moiety			
1″	4.95 (d, 7.6)	101.9	C-5′
2″	3.604 (dd, 1.6, 7.2)	74.6	
3″	3.53 (m)	77.3	
4″	3.46 (d, 4.8)	71.2	
5″	3.46 (d, 4.8)	78.2	
6″	3.73 (m, H-6′ a)	62.3	
	3.92 (m, H-6′ b)		

**Table 2:** Antioxidant activities of the isolated compounds (**1**–**6**) from *F. tuberculosa*.

Compound No.	Compound Name	IC_50_ Value (µg/mL)	
		ABTS Assay	FRAP Assay
1	Phloroglucinol	6.44 ± 0.47	12.89 ± 0.93
2	Phlorin	43.40 ± 3.18	64.52 ± 4.15
3	Isorhamnetin 3-*O*-rutinoside	12.24 ± 0.61	25.24 ± 2.03
4	(-) Catechin-(2→1′,4→2′)-phloroglucinol	4.11 ± 0.32	7.36 ± 0.57
5	Isorhamnetin 3-*O*-[*α*-rhamnopyranosyl-(1→4)-*α*-rhamnopyranosyl-(1→6)-*β*-glucopyranoside]	18.19 ± 2.94	35.04 ± 2.89
6	*β*-sitosterol	557.46 ± 19.76	ND
	Positive standard (Ascorbic acid)	10.67 ± 0.85	20.86± 1.28

All values are expressed as mean ± SD (*n* = 3).

**Table 3:** Molecular docking results for compounds phlorin (**2**) and isorhamnetin 3-*O*-rutinoside (**3**) during docking in the tyrosinase (PDB ID: 2Y9X) active domain (using kojic acid as reference drug) and the hyaluronidase (PDB ID: 1FCV) active site (using luteolin as reference drug), including binding affinities (kcal/mol), distance (Å) from main residue/metal contact, and the type of the interaction.

Tyrosinase (PDB ID: 2Y9X)				
Compound	Affinity (kcal/mol)	Distance (in Å) from Main Residue		Type of Interaction
Phlorin	−10.935	2.61	Cu400	Metal acceptor
		2.55	Cu401	Metal acceptor
		2.50	His85	H-bond acceptor
		2.82	Asn260	H-bond donor
		2.74	Met280	H-bond donor
		4.93	His244	Hydrophobic
Isorhamnetin 3-*O*-rutinoside	−12.154	2.12	Cu400	Metal acceptor
		2.26	Cu401	Metal acceptor
		2.36	His85	H-bond acceptor
		2.97	Met280	H-bond donor
		2.27	Val283	H-bond acceptor
		2.53	Asn81	H-bond acceptor
		3.14	His263	Hydrophobic
		3.45	Gly281	Hydrophobic
kojic acid	−9.385	2.96	Cu400	Metal acceptor
		2.63	Cu401	Metal acceptor
		2.34	His85	H-bond acceptor
		2.90	Asn260	H-bond acceptor
		2.78	Met280	H-bond donor
		3.89	His263	Hydrophobic
		3.39	Val283	H-bond acceptor
Hyaluronidase (PDB ID: 1FCV)				
Compound	Affinity (kcal/mol)	Distance (in Å) from Main Residue		Type of Interaction
Phlorin	−14.979	1.91	Asp111	H-bond donor
		2.34	Ser304	H-bond acceptor
		1.93	Glu113	H-bond donor
		2.98	Glu113	H-bond donor
		4.11	Trp301	Hydrophobic
		2.51	Tyr184	H-bond acceptor
Isorhamnetin 3-*O*-rutinoside	−16.578	2.15	Asp111	H-bond donor
		2.89	Ser304	H-bond acceptor
		2.36	Ser303	H-bond acceptor
		2.41	Ser303	H-bond acceptor
		1.99	Glu113	Electrostatic
		3.14	Trp301	Hydrophobic
		3.54	Tyr55	Hydrophobic
		3.22	Asp305	Hydrophobic
Luteolin	−12.404	2.66	Asp111	H-bond donor
		2.00	Ser304	H-bond acceptor
		3.57	Glu113	Hydrophobic
		4.74	Trp301	Hydrophobic
		4.77	Tyr55	Hydrophobic
		2.45	Asp56	H-bond acceptor
		1.95	Asp305	H-bond donor

## Data Availability

All data generated or analyzed during this study are available in this published article and the provided supplementary materials.

## Supplementary Materials

The following supporting information can be downloaded at: https://www.mdpi.com/article/10.3390/molecules28196895/s1, Figure S1: ^1^H NMR spectrum of compound **1** (400 MHz, *CD*_3_*OD*); Figure S2: DEPT-Q spectrum of compound **1** (100 MHz, *CD*_3_*OD*); Figure S3: ^1^H NMR spectrum of compound **2** (400 MHz, *CD*_3_*OD*); Figure S4: DEPT-Q spectrum of compound **2** (100 MHz, *CD*_3_*OD*); Figure S5: HMBC spectrum of compound **2**; Figure S6: ^1^H NMR spectrum of compound **3** (400 MHz, *CD*_3_*OD*); Figure S7: Negative-mode HR-ESI-MS spectrum of compound **4**; Figure S8: ^1^H NMR spectrum of compound **4** (400 MHz, *CD*_3_*OD*); Figure S9: DEPT-Q spectrum of compound **4** (100 MHz, *CD*_3_*OD*); Figure S10: HSQC spectrum of compound **4**; Figure S11: HMBC spectrum of compound **4**; Figure S12: NOESY spectrum of compound **4**; Figure S13: Expanded NOESY spectrum of compound **4**; Figure S14: ^1^H NMR spectrum of compound **5** (400 MHz, *CD*_3_*OD*); Figure S15: DEPT-Q spectrum of compound **5** (100 MHz, *CD*_3_*OD*).

## Sample Availability

Not available.
