# Supplementary material for: Antioxidant and Anti-Aging Phytoconstituents from Faucaria tuberculosa: In Vitro and In Silico Studies
Source: Molecules. 2023 Sep 30;28(19):6895. doi: 10.3390/molecules28196895 (PMC10574154; doi:10.3390/molecules28196895)
Supplement: Supplementary file 1 [file molecules-28-06895-s001.zip › molecules-2613968-supplementary.pdf]

Supplementary Material

Antioxidant and Anti-aging Phytoconstituents from *Faucaria tuberculosa*: *in vitro* and in *silico* studies

Hayam S. Ahmed[1#], Hala Abouzeid[2#], Mostafa A. Mansour[3], Asmaa I. Owis[1,4], Elham Amin[1,5*], Hany W. Darwish [6], Ashwag S. Alanazi [7], Ibrahim A. Naguib [8], Naglaa Afifi[1*]

[1]Department of Pharmacognosy, Faculty of Pharmacy, Beni-Suef University, Beni-Suef 62514, Egypt.

[2]Department of Pharmacognosy, Faculty of Pharmacy, Port Said University, Port Said 42515, Egypt

[3]Department of pharmaceutical chemistry, Faculty of Pharmacy, Nahda University, Beni-suef, Egypt.

[4]Department of Pharmacognosy, Faculty of Pharmacy, Heliopolis University for Sustainable Development, Cairo 11785, Egypt.

[5]Department of Medicinal Chemistry and Pharmacognosy, College of Pharmacy, Qassim University, Buraydah 51452, Saudi Arabia.

[6] Department of Pharmaceutical Chemistry, College of Pharmacy, King Saud University, Riyadh 11451, Saudi Arabia.

[7]Department of Pharmaceutical Sciences, College of Pharmacy, Princess Nourah bint Abdulrahman University, Riyadh 11671, Saudi Arabia.

[8] Department of Pharmaceutical Chemistry, College of Pharmacy, Taif University, P.O. Box 11099, Taif 21944, Saudi Arabia.

Abstract

The research targeting natural cosmeceuticals is now increasing due to the safety and/or limited side effects of natural products that are highly valued in cosmetology. In a research program aimed at exploring botanical sources for valuable skin-care antioxidant components, the current study investigated the phytochemical content and the biological potential of *Faucaria tuberculosa*. Phytochemical investigation of *F. tuberculosa* extract resulted in purification and characterization of six phytoconstituents including one new. The structure of the new constituent was elucidated as catechin-(2→1′,4→2′)-phloroglucinol **(4)**. The structural identity of all isolated compounds were confirmed on the basis of extensive physical and spectral (1D, 2D-NMR and HRESIMS) investigations. The ethanolic extract exhibits a rich content of total phenolics (TPC) and total flavonoids (TFC) estimated as 32±0.034 mg GAE/g and 43± 0.004 mg RE/g, respectively. In addition, the antioxidant (ABTS and FRAP), antihyaluronidase and the antityrosinase activities of

all purified phytoconstituents were evaluated. The results noted phloroglucinol **(1)** and catechin-(2→1′,4→2′)-phloroglucinol (**4**) for remarkable antioxidant activity. While on the other side 3,5-dihydroxyphenyl *β*-D-glucopyranoside (**2**) achieved the most potent inhibitory activity against tyrosinase and hyaluronidase enzymes (IC_{50} 8.637 ± 0.44 µg/ml and 18.04 ± 0.92 µg/ml, respectively) that was comparable to the standard drugs kojic acid (IC_{50} 9.267 µg/ml) and luteolin, (IC_{50} 33.25 ± 1.69 µg/ml). Molecular docking study of 3,5-dihydroxyphenyl *β*-D-glucopyranoside (2) highlights its high potential to bind to the active sites of two enzymes involved in the study.

Table of Contents

No	Content	Page
**S1**	^{1}H NMR spectrum of compound **1** (400 MHz, *CD_3OD*).	**S-3**
**S2**	DEPT-Q spectrum of compound **1** (100 MHz, *CD_3OD*).	**S-4**
**S3**	^{1}H NMR spectrum of compound **2** (400 MHz, *CD_3OD*).	**S-5**
**S4**	DEPT-Q spectrum of compound **2** (100 MHz, *CD_3OD).*	**S-6**
**S5**	HMBC spectrum of compound **2**	**S-7**
**S6**	^{1}H NMR spectrum of compound **3** (400 MHz, *CD_3OD).*	**S-8**
**S7**	Negative mode HR-ESI- MS spectrum of compound **4**	**S-9**
**S8**	^{1}H NMR spectrum of compound **4** (400 MHz*, CD_3OD)*	**S-10**
**S9**	DEPT-Q spectrum of compound **4** (100 MHz*, CD_3OD).*	**S-11**
**S10**	HSQC spectrum of compound **4**	**S-12**
**S11**	HMBC spectrum of compound **4**	**S-13**
**S12**	NOESY spectrum of compound **4**	**S-14**
**S13**	Expanded NOESY spectrum of compound **4.**	**S-15**
**S14**	^{1}H NMR spectrum of compound **5** (400 MHz*, CD_3OD).*	**S-16**
**S15**	DEPT-Q spectrum of compound **5** (100 MHz*, CD_3OD).*	**S-17**

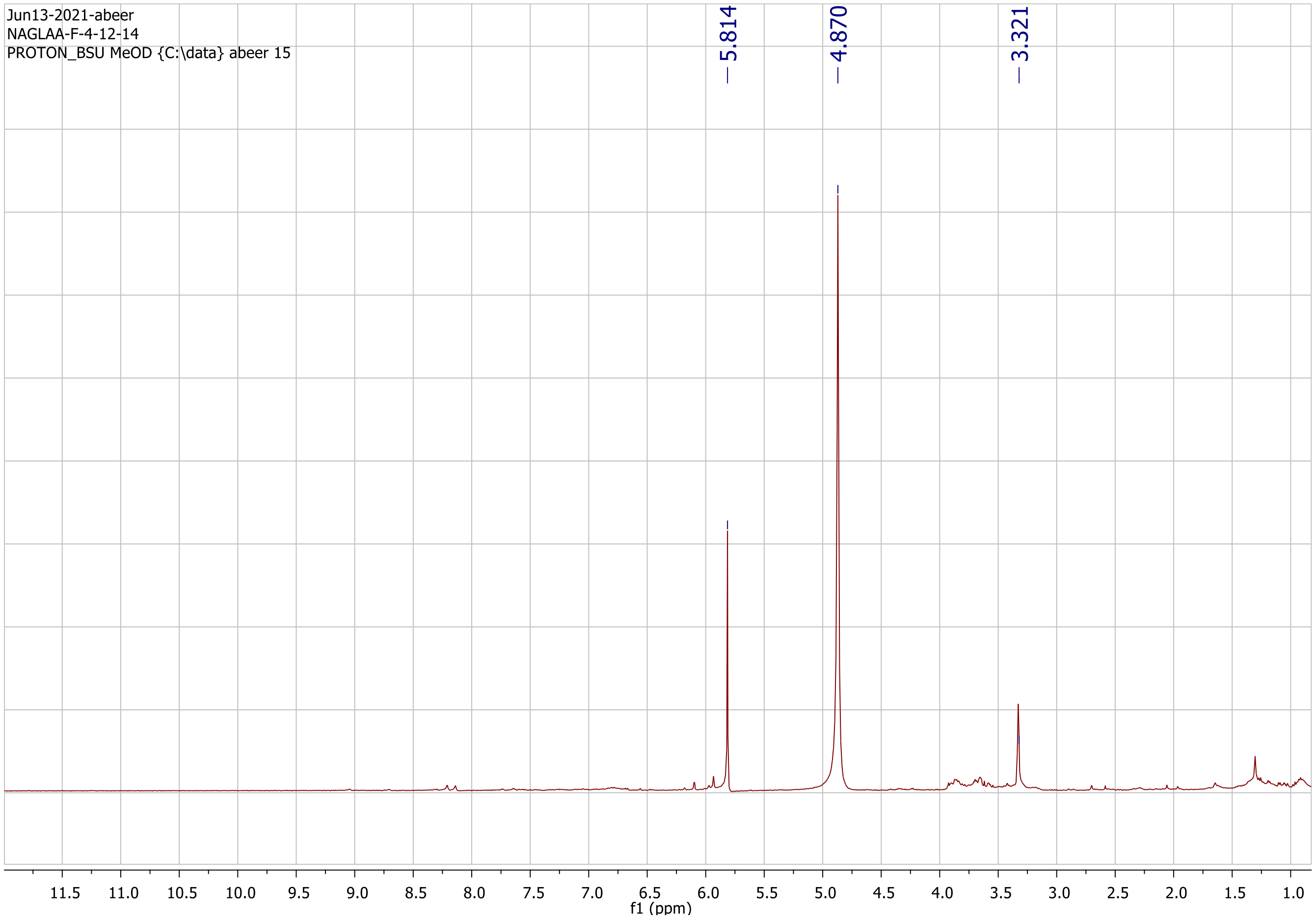
Jun13-2021-abeer
NAGLAA-F-4-12-14
PROTON_BSU MeOD {C:\data} abeer 15
5.814
4.870
3.321
11.5 11.0 10.5 10.0 9.5 9.0 8.5 8.0 7.5 7.0 6.5 6.0 5.5 5.0 4.5 4.0 3.5 3.0 2.5 2.0 1.5 1.0
f1 (ppm)

Figure S1: [1] H NMR spectrum of compound 1 (400 MHz, CD_3OD).

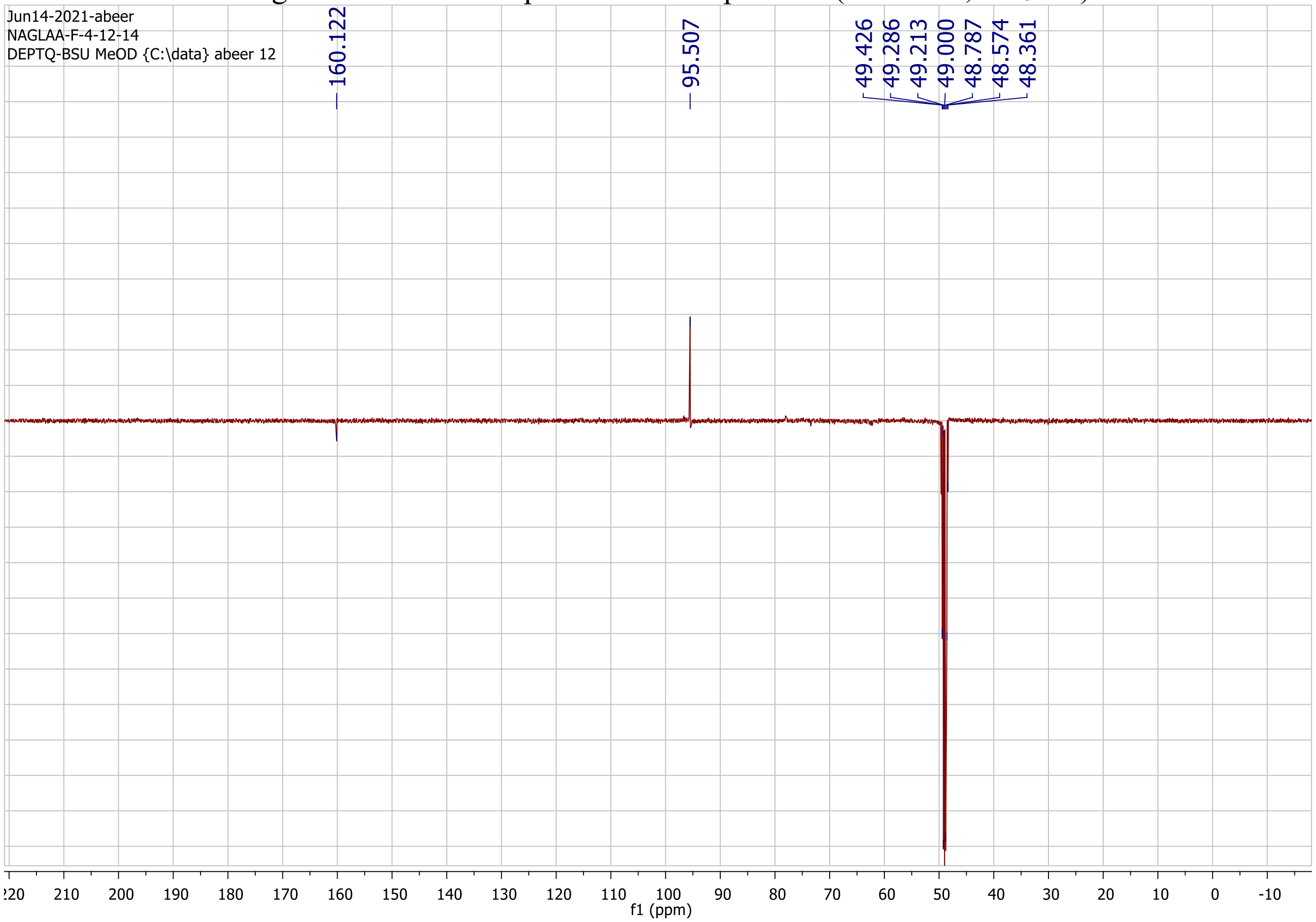

Figure S2: DEPT-Q spectrum of compound 1 (100 MHz, CD_3OD).

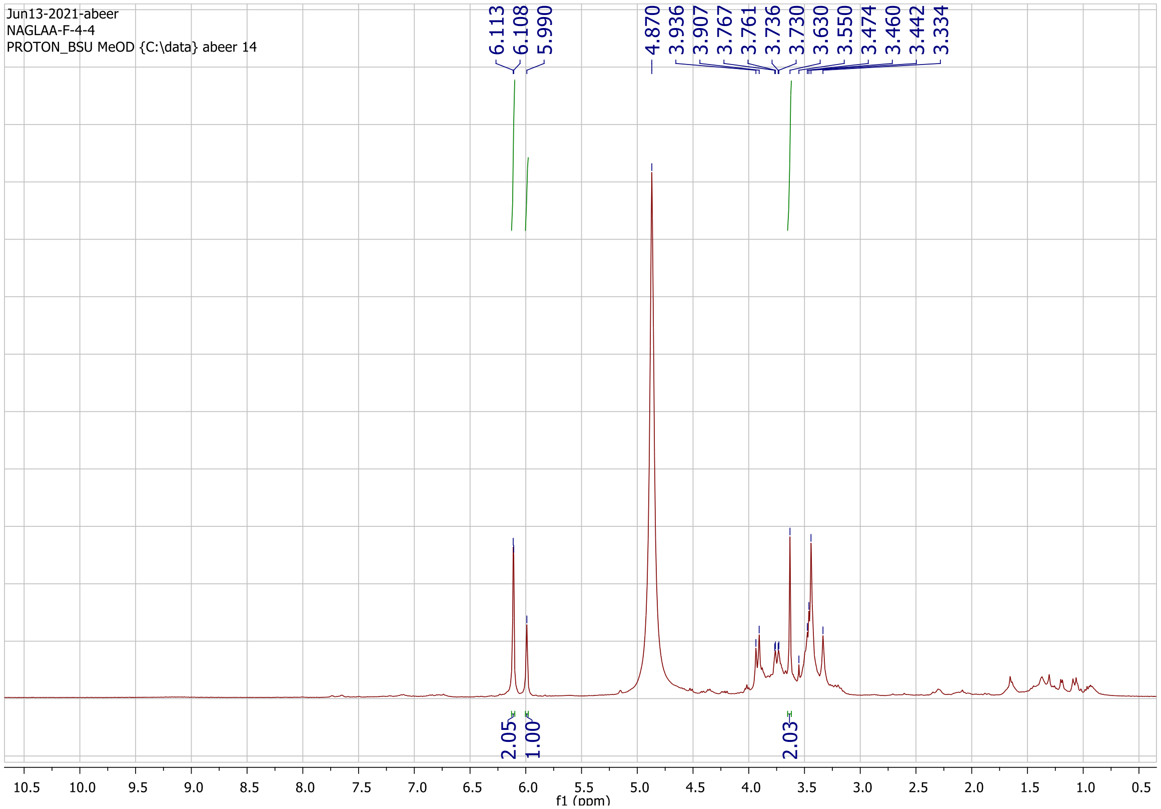

Figure S3: [1] H NMR spectrum of compound 2 (400 MHz, *CD_3OD*).

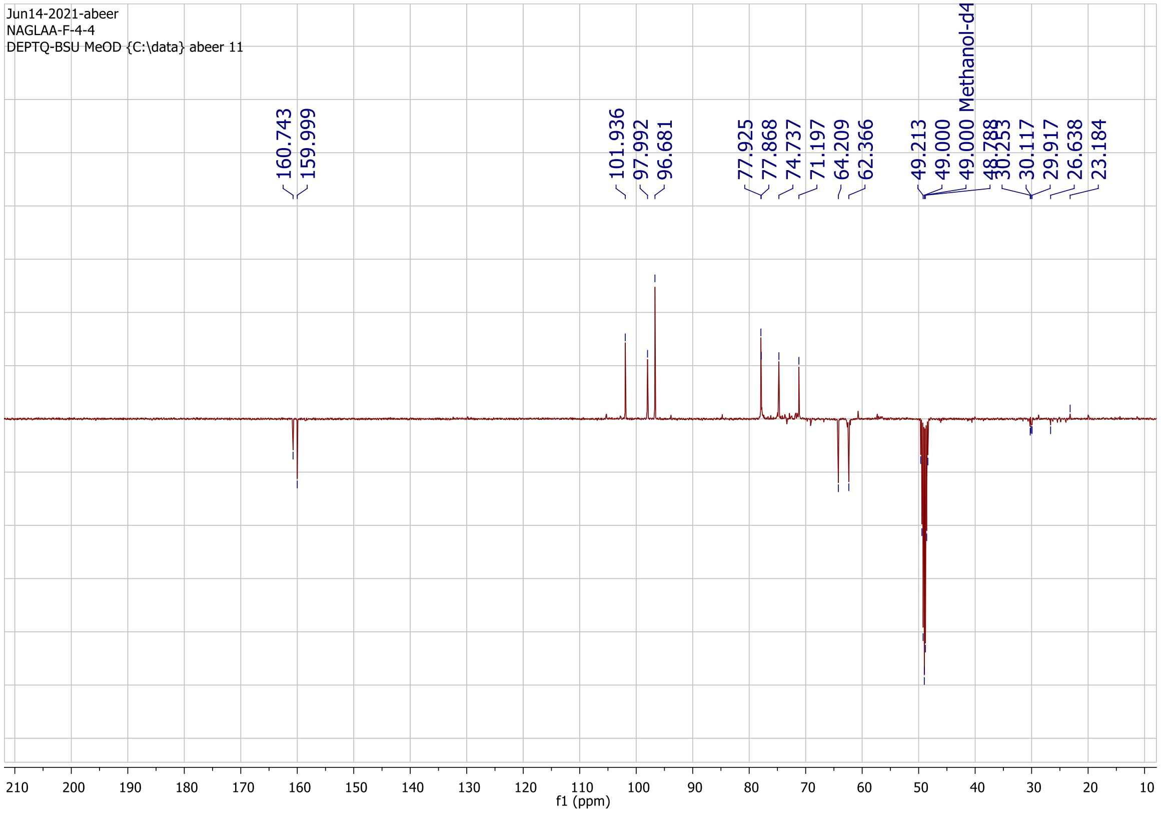

Figure S4: DEPT-Q spectrum of compound **2** (100 MHz, CD_3OD).

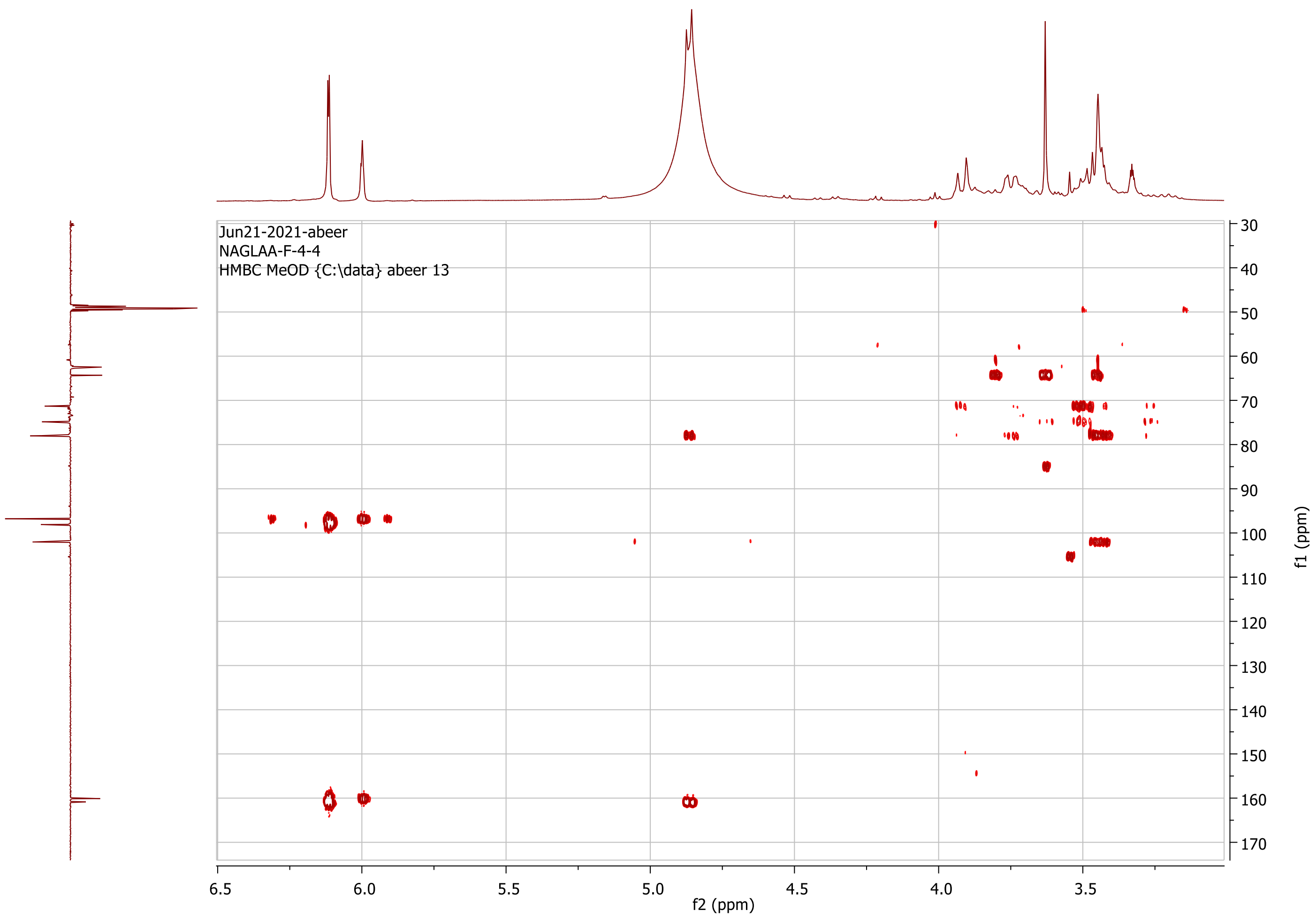

Figure S5: HMBC spectrum of compound **2**

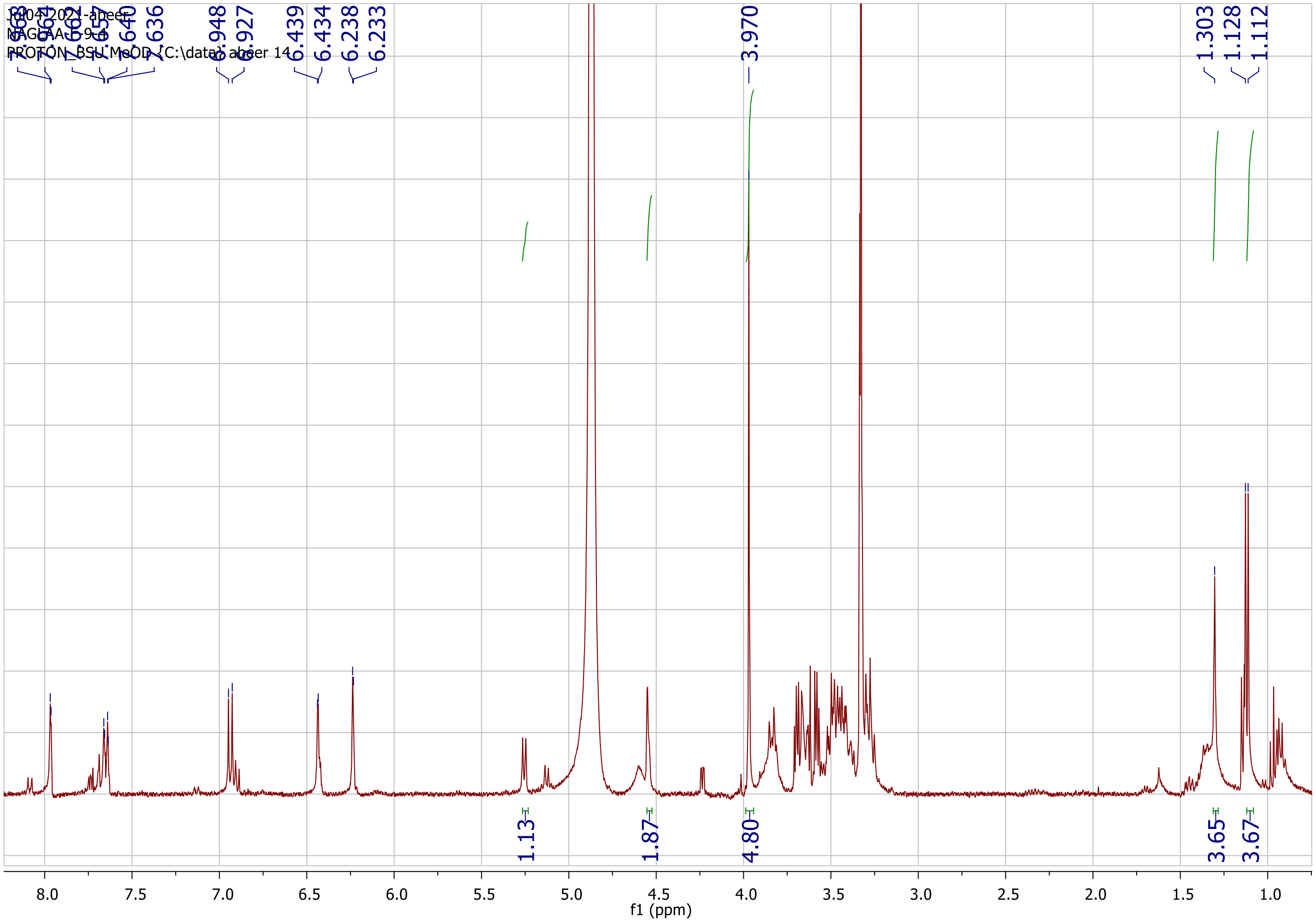

Figure S6: [1] H NMR spectrum of compound 3 (400 MHz, CD_3OD).

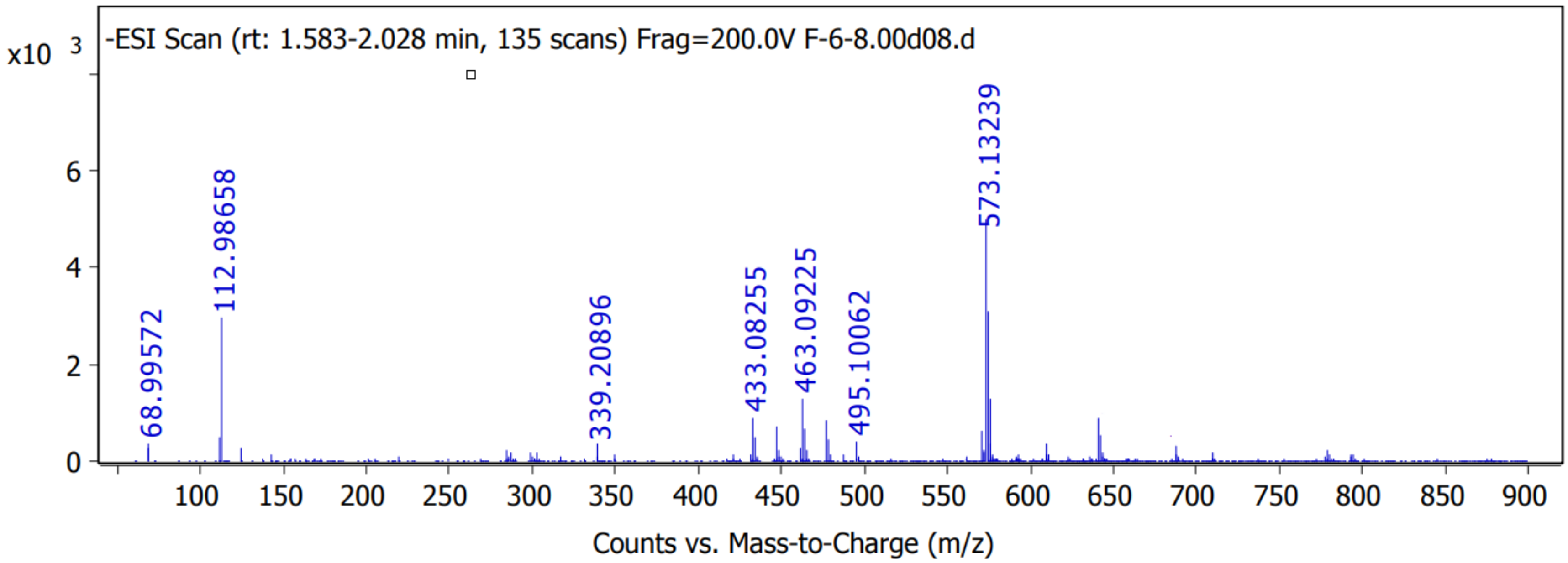

Figure S7: Negative mode HR-ESI- MS spectrum of compound **4**

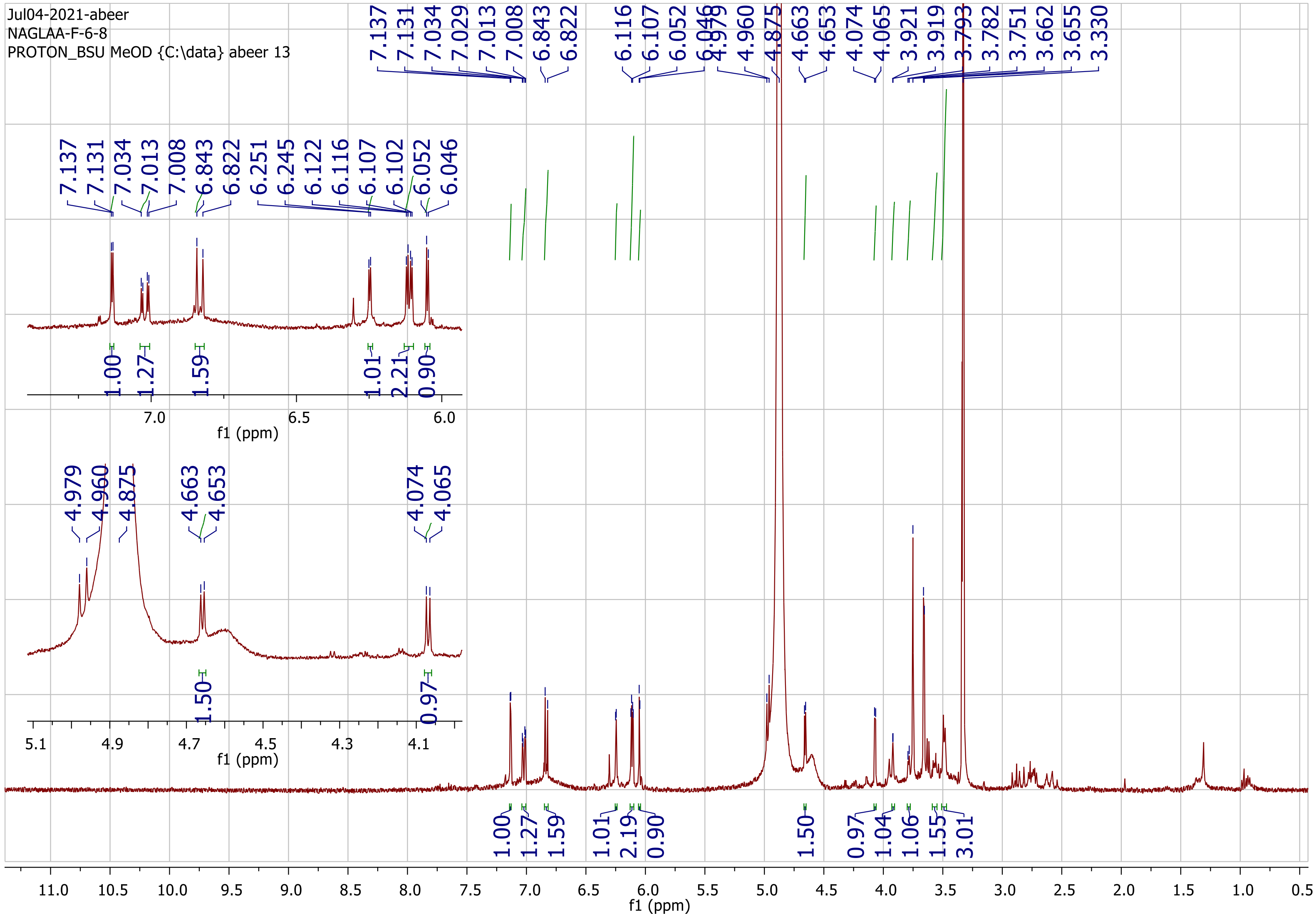

Figure S8: ^{1}H NMR spectrum of compound 4 (400 MHz, CD_3OD).

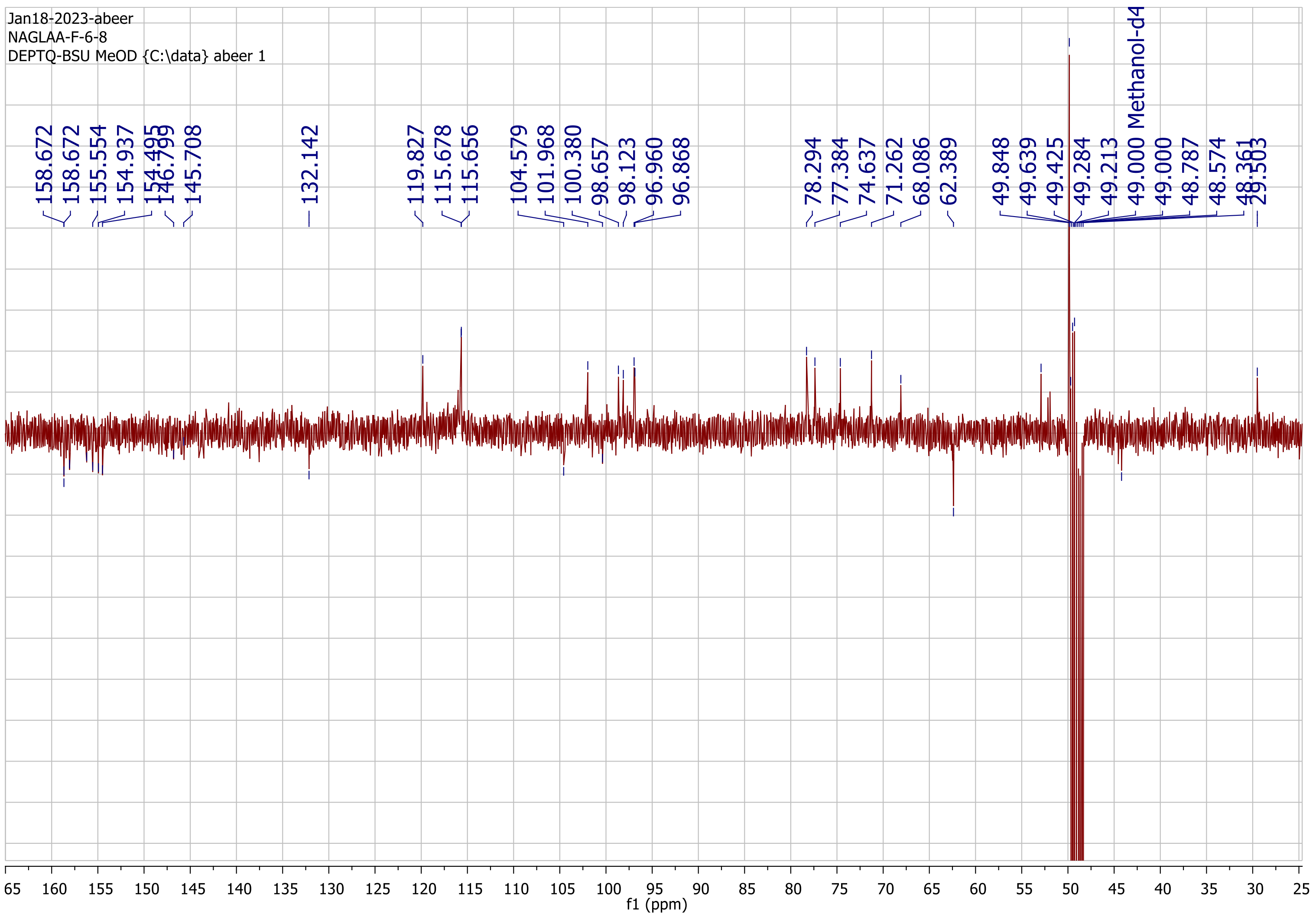

Figure S9: DEPT-Q spectrum of compound **4** (100 MHz, CD_3OD).

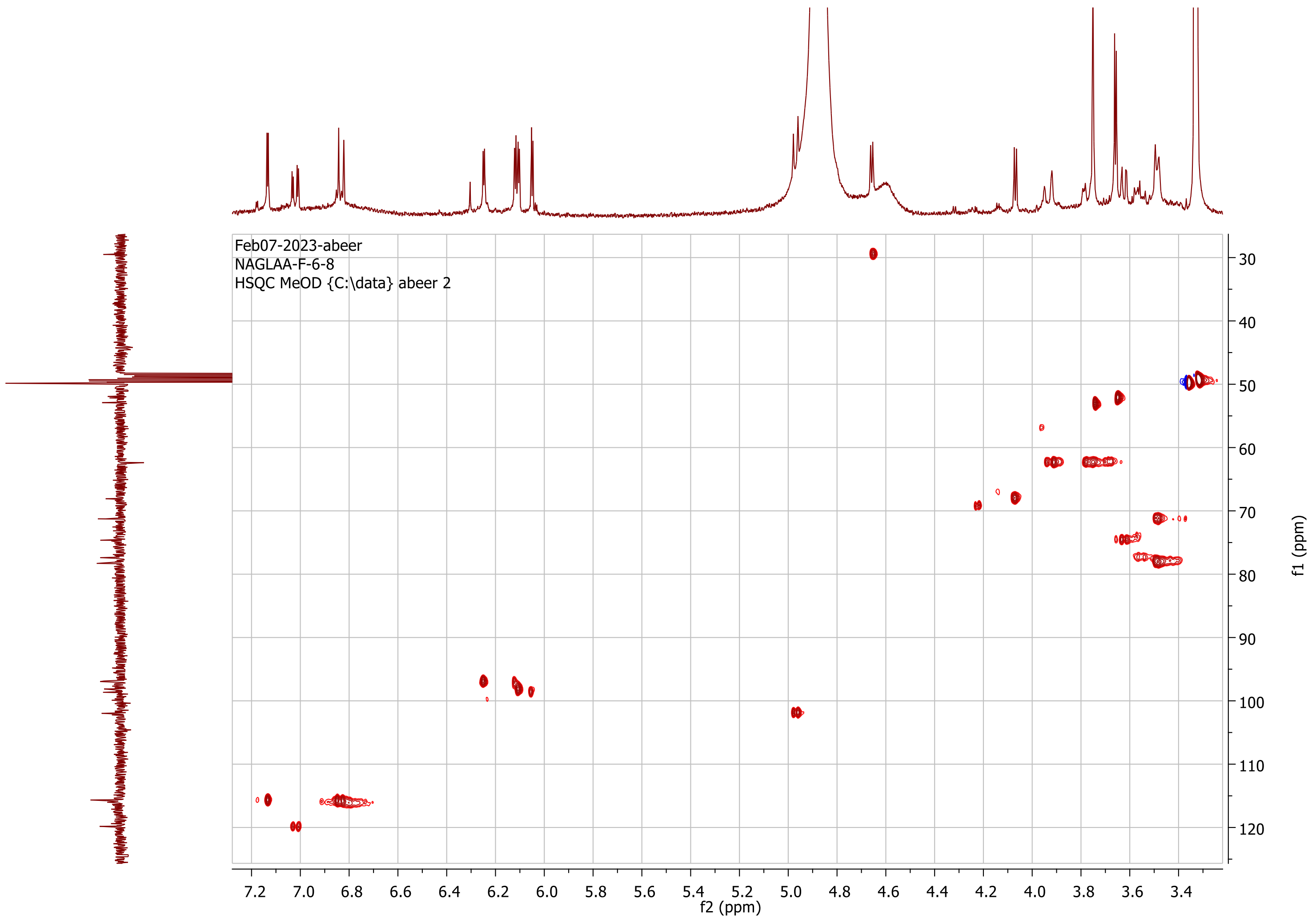

Figure S10: HSQC spectrum of compound **4**

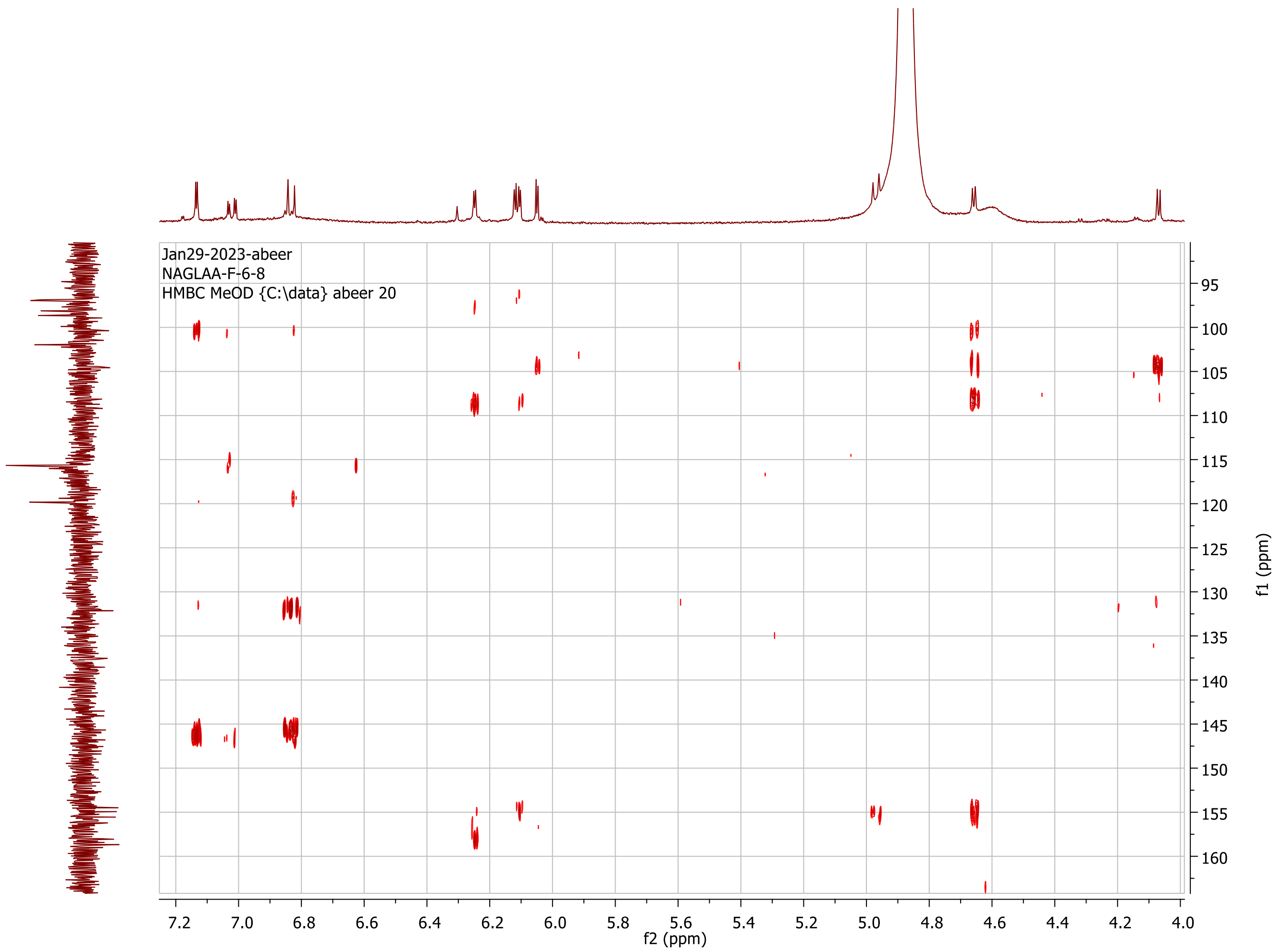

Figure S11: HMBC spectrum of compound **4**

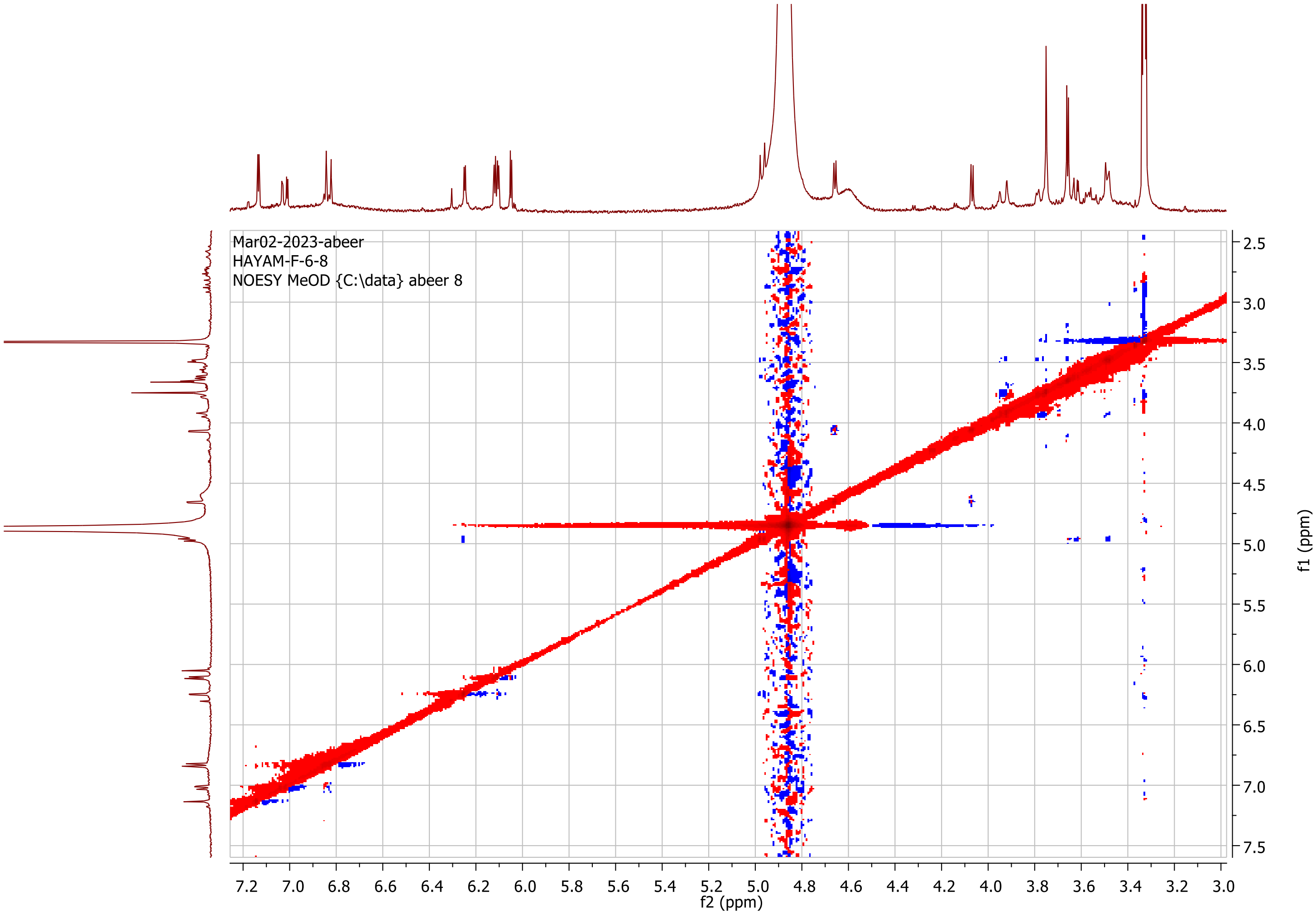

Figure S12: NOESY spectrum of compound **4**

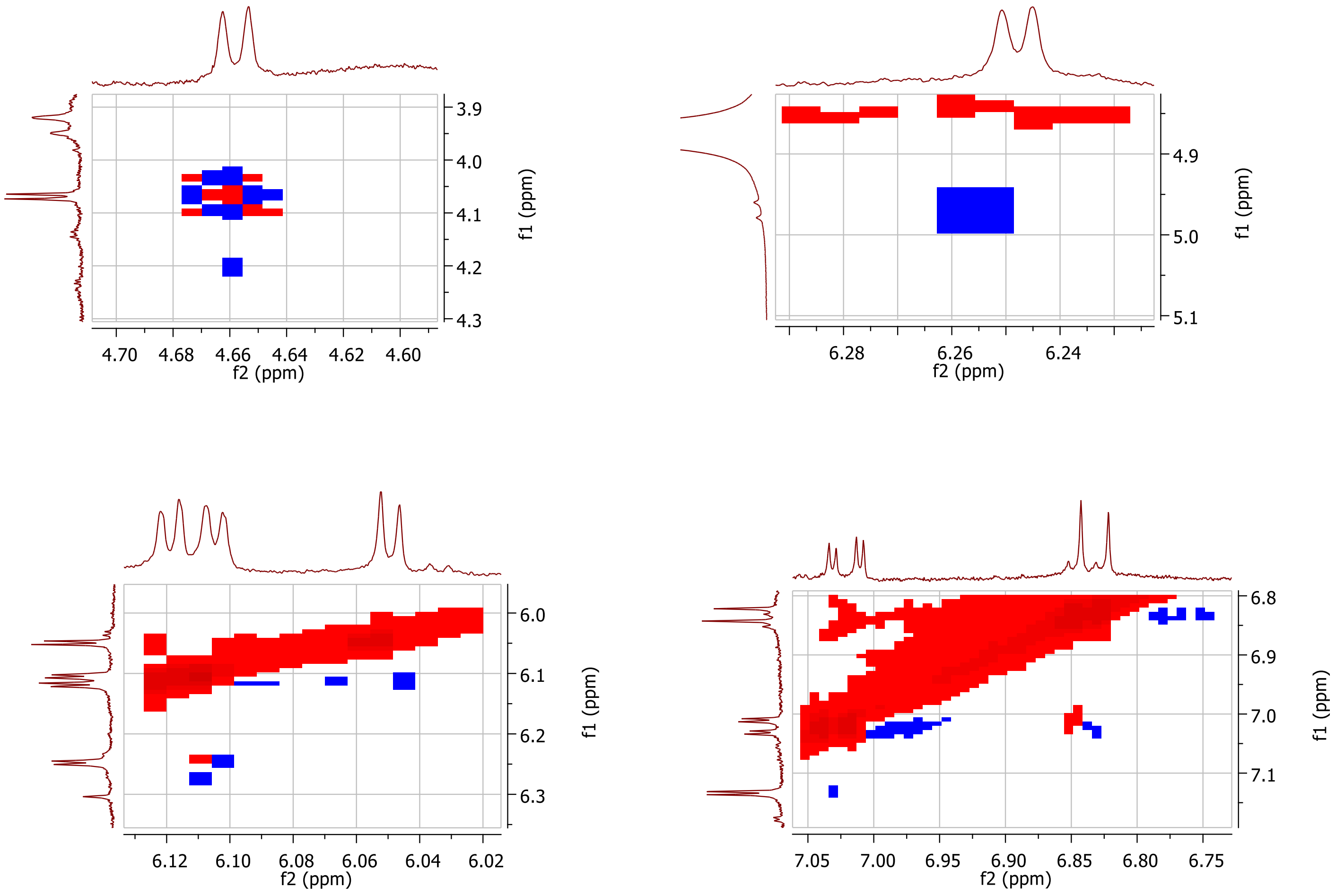

Figure S13: Expanded NOESY spectrum of compound **4**

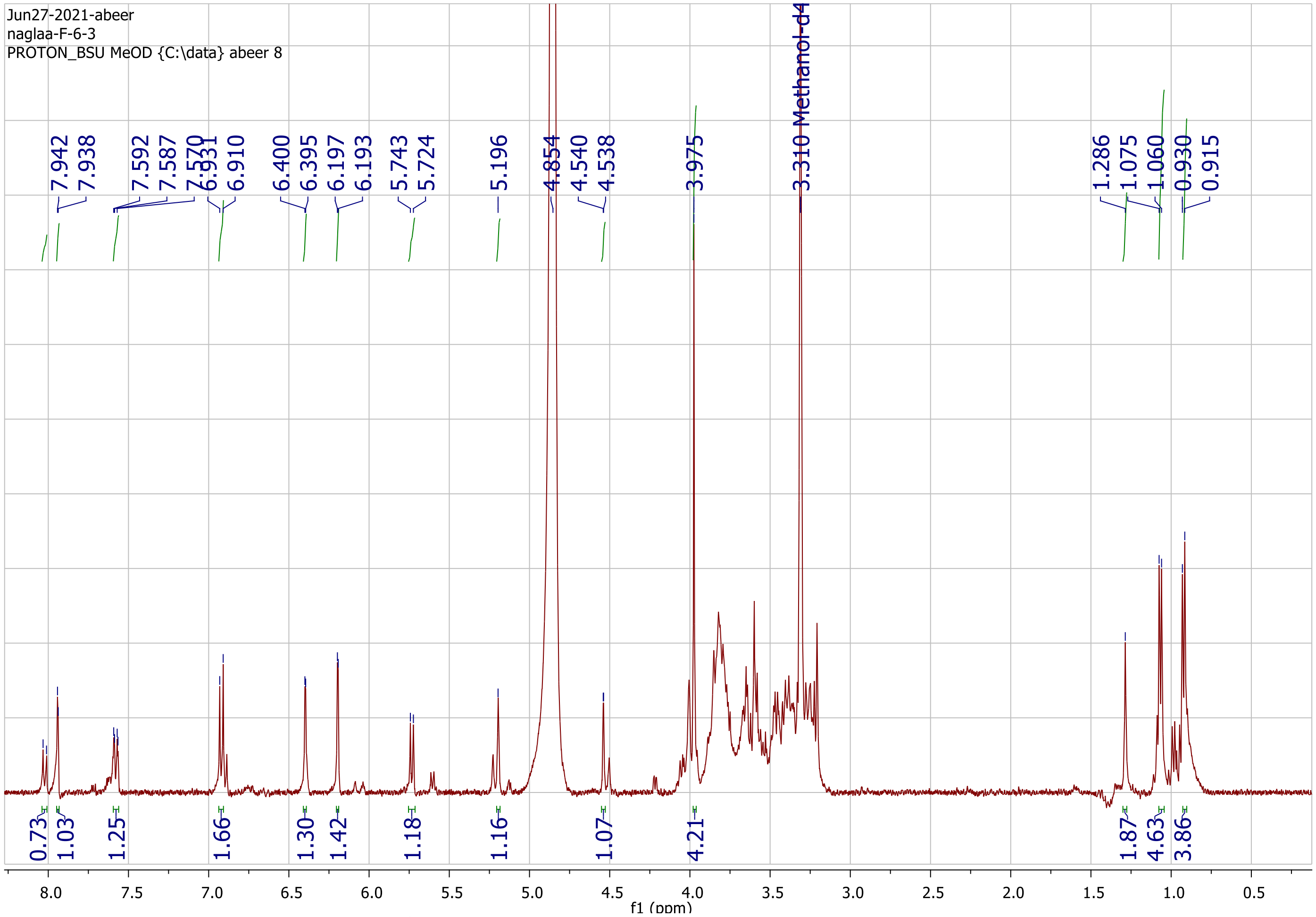

Figure S14: [1] H NMR spectrum of compound 5 (400 MHz, CD_3OD).

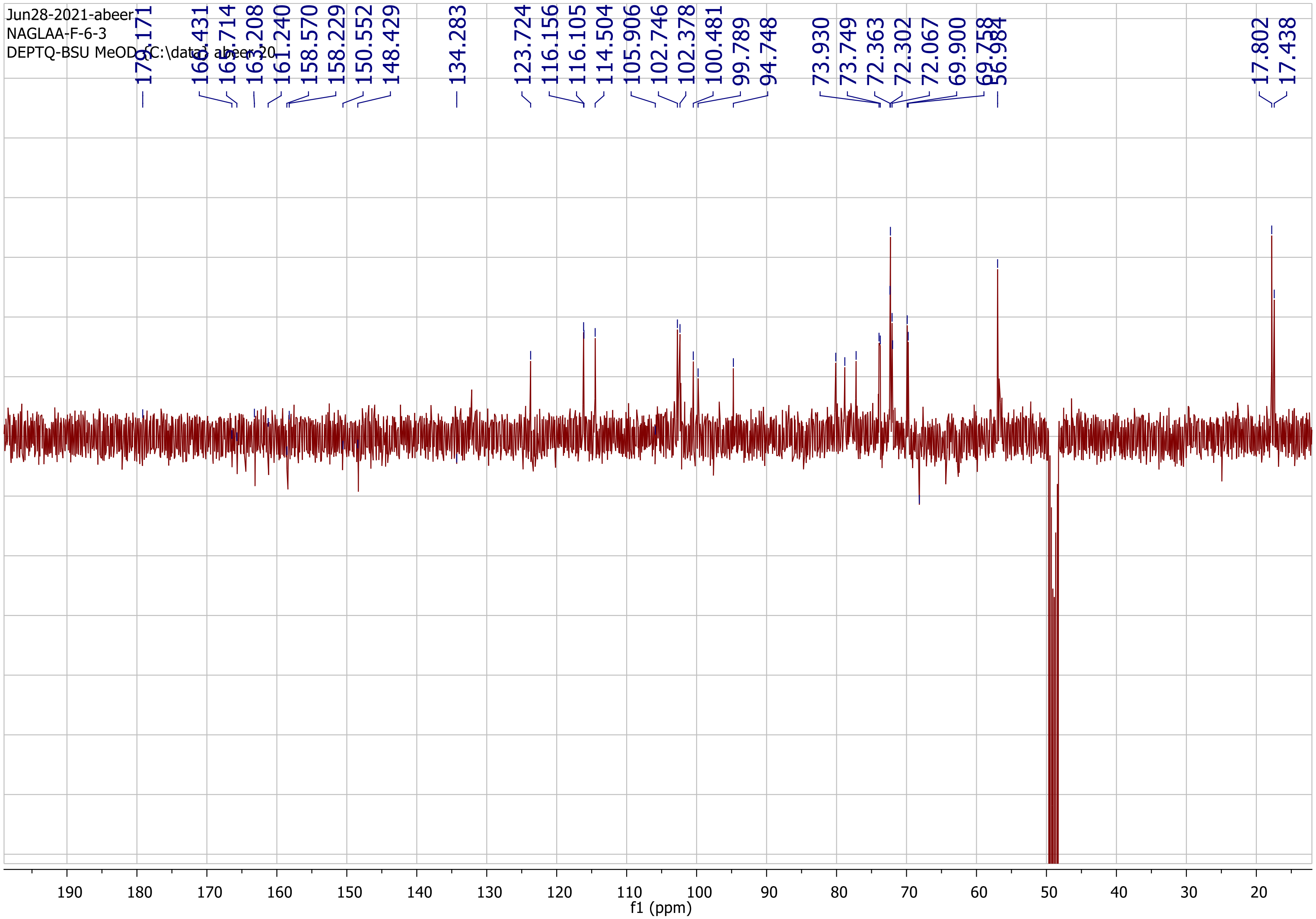

Figure S15: DEPT-Q spectrum of compound 5 (100 MHz, CD_3OD).

NMR data for compounds: **1, 2,3, 5** and **6** were included in supplementary data

Compound 1: Phloroglucinol

^{1}H-NMR (400 MHz, MeOD-*d*4); δ_H 5.81 (3H, s, H-2, H-4, H-6. DEPT-Q NMR (101 MHz, MeOD-*d*4); δ_C 95.5 (C-1, C-3, C-5), 160.12(C-2, C-4, C-6) (Figure S1 and S2).

Compound 2: 3,5-dihydroxyphenyl *β*-D-glucopyranoside (Phlorin); ^{1}H-NMR (400 MHz, MeOD-*d*4); δ_H 6.09 (2H, d, 2, H-2 and H-6), 5.97 (1H, t, 2, H-4), 4.86 (1H, d, 7.6 Hz, H-1′), 3.61-3.42 (4H, m, H-2′,3′,4′,5′), 3.72 (1H, dd, 12.1 and 2.4 Hz, H-6′ a), 3.90 (1H, d, 11.6, H-6′ b); DEPT-Q NMR (101 MHz, MeOD-*d*4); δ_C 160.7 (C-1), 159.99 (C-3 and C-5), 97.9 (C-4), 96.6 (C-2 and C-6), 101.9 (C-1′), 77.9 (*d*, C-5′), 77.8 (C-3′), 74.7 (C-2′), 71.2 (C-4′), 62.3 (C-6′) (Figure S3 : S5).

Compound 3: isorhamnetin 3-*O*-rutinoside; ^{1}H-NMR (400 MHz, MeOD-*d*4); δ_H 7.94 (1H, d, 1.6, H-2′), 7.63 (1H, dd, 1.6, 8.4, H-6′), 6.91 (1H, d, 8.4, H-5′), 6.41 (1H, d, 2, H-8), 6.21 (1H, d, 2, H-6), 5.23 (1H, d, 7.6, H-1″), 4.53 (1H, br s, H-1‴), 3.2 - 3.7 (H-2″-H-6″, H-2‴-H-5‴), 1.09 (3H, d, 6.4, H-6‴) (Figure S6).

Compound 5: Isorhamnetin 3-*O*-[*α*-rhamnopyranosyl-(1-4)-α-rhamnopyranosyl-(1-6)-*β*-glucopyranoside]; ^{1}H-NMR (400 MHz, MeOD-*d*4); δ_H 7.94 (1H, d, 1.6, H-2′), 7.59 (1H, dd, 2, 8.8 , H-6′), 6.92 (1H, d, 8.4, H-5′), 6.39 (1H, d, 2, H-8), 6.19 (1H, d, 2, H-6), 5.73 (1H, d, 7.6, H-1″), 5.19 (1H, br s, H-1‴), 4.54 (1H, br s, H-1⁗), 3.97 (3H, s, OCH_3), 3.00 - 4.00 (H-2″- H-6″, H-2‴-H-5‴, H- 2⁗-H-5⁗), 1.06 (3H, d, 6, H-6‴), 0.92 (3H, d, 6, H-6⁗). Multiplicities of most sugar resonances were not determined, because of signal overlap. DEPT-Q NMR (101 MHz, MeOD-*d*4); δ_C 158.5 (C-2), 134.2 (C-3), 179.2 (C-4), 163.2 (C-5), (99.8) (C-6), 166.4(C-7), 94.7 (C-8), 158.2 (C-9), 105.9 (C-10), 123.3 (C-1′), 114.5 (C-2′), 150.6 (C-3′), 148.3 (C-4′), 116.2 (C-5′), 123.7 (C-6′) , 56.9 (OCH_3), 102.7 (C-1″), 73.9 (C-2″), 78.8 (C-3″), 72.0 (C-4″), 77.2 (C-5″), 68.1 (C-6″), 102.3 (C-1‴), 72.3 (C-2‴), 72.3 (C-3‴), 80.1 (C-4‴), 69.8(C-5‴), 17.8 (C-6‴), 100.4 (C- 1⁗), 72.1(C-2⁗), 72.4(C- 3⁗), 73.8 (C-4⁗), 69.9 (C-5⁗), 17.5(C-6⁗) (Figure S14 and S15).

Compound 6: *β*-sitosterol; ^{1}H NMR (400 MHz, $CDCl_3$) signals; δ_H: 5.36 (d, *J* = 8 Hz, H-6), 3.52 (m, H-3), 1.01 (CH_3-19), 0.93 (CH_3-21), 0.84 (CH_3-26), 0.83 (CH_3-27), 0.81 (CH_3-29), and 0.68 (CH_3-18); DEPT-Q NMR (101 MHz, $CDCl_3$) δ_C: 37.2 (C-1), 31.6 (C-2), 71.8 (C-3), 42.2 (C-4), 140.7 (C-5), 121.7 (C-6), 31.9 (C-7), 31.9 (C-8), 50.1 (C-9), 36.5 (C-10), 21.06 (C-11), 39.7 (C-12), 42.19 (C-13), 56.8 (C-14), 24.4 (C-15), 28.9 (C-16), 55.9 (C-17), 12.04 (C-18), 19.5 (C-19), 36.1 (C-20), 18.9 (C-21), 33.9 (C-22), 26.2 (C-23), 45.8 (C-24), 29.3 (C-25), 20.0 (C-26), 19.2 (C-27), 23.3 (C-28), and 12.3 (C-29).
